# Biomechanical anticoagulation by spherical platelets in extracorporeal systems

**DOI:** 10.1073/pnas.2535113123

**Published:** 2026-03-30

**Authors:** Rui Ji, Yongjian Li, Jiang Li, Haosheng Chen

**Affiliations:** ^a^State Key Lab of Tribology, Department of Mechanical Engineering, Tsinghua University, Beijing 100084, China; ^b^Department of Mechanical Engineering, University of Science and Technology Beijing, Beijing 100083, China

**Keywords:** friction, anticoagulation, integrin, platelet activation, hemodialysis

## Abstract

Extracorporeal life support, such as hemodialysis, often face the risk of blood coagulation, where systemic anticoagulation with heparin is routinely administered. However, this strategy is contraindicated in patients at high risk of bleeding. Inspired by the observation that rolling motion of spherical platelets avoids their exposure to tangential forces, an anticoagulation strategy is proposed based on platelet spherification for extracorporeal systems. Experimental results prove that spherical platelets retain activation pathways and capacity compared to discoid platelets. Mechanical activation is driven by tangential rather than normal forces, and maintaining rolling instead of sliding effectively prevents tangential force-induced activation. Molecular dynamics simulations reveal that tangential force acting on sliding discoid platelets separates the integrin *α_IIb_β_3_* head from the lower leg region to unfold and activate the integrin, while the normal force acting on rolling spherical platelets induces headpiece rotation without triggering unfolding, which effectively reduces integrin *α_IIb_β_3_* activation. In in vitro hemodialysis experiments, platelet spherification effectively minimized coagulation, while avoiding bleeding complications.

## Significance

Extracorporeal life support currently relies on systemic anticoagulant drugs to keep circuits patent, but these drugs inevitably increase bleeding risk. The need to prevent thrombosis without increasing bleeding risk remains a major challenge. This work exploits platelet spherification, a reversible change from discoid to spherical shape, to prevent thrombosis while preserving hemostatic potential. Spherical platelets roll rather than slide along artificial surfaces, diminishing tangential forces that mechanically activate integrin *α_IIb_β_3_* and drive thrombus growth, while retaining platelet responses to soluble agonists. This biomechanical approach links platelet mechanobiology to a clinically actionable strategy for anticoagulation in blood-contacting devices.

Extracorporeal life support, including hemodialysis (HD), continuous renal-replacement therapy (CRRT), and extracorporeal membrane oxygenation (ECMO), inevitably exposes blood to artificial surfaces, thereby predisposing to thrombotic complications (1). In clinical treatments, anticoagulation is most commonly achieved with systemic heparin, regional citrate anticoagulation (RCA), or other oral anticoagulants (2). Systemic heparin is associated with a bleeding event rate of 8.5% (3) and a cumulative heparin-induced thrombocytopenia (HIT) risk of 12% (4). RCA avoids HIT, but it requires continuous calcium supplementation and is limited by metabolic alkalosis, especially in patients with hepatic impairment or hemodynamic instability (5). Therefore, in extracorporeal life support, there is an urgent demand for other alternative therapeutic strategies that not only mitigate the risk of coagulation but also reduce the necessity for anticoagulant medications (6).

Platelet activation is a fundamental step in the coagulation process and can be initiated through two major pathways: mechanical stimulation and chemical agonists (7). Both pathways converge on the activation of integrin *α*_IIb_*β*_3_, promoting platelet aggregation and thrombus formation. When circulating platelets approach the surface, their movement is slowed by frictional forces arising from the binding of platelet integrin *α*_IIb_*β*_3_ to surface-adsorbed ligands (8). A well-studied example is the bond between integrin *α*_IIb_*β*_3_ to its ligands, including fibrinogen or von Willebrand factor (vWF) (9), which ultimately leads to irreversible platelet adhesion. Traditionally, integrin–ligand bonding forces are simplified as normal forces (10–13). However, integrin *α*_IIb_*β*_3_ activation represents an anisotropic mechanosensory process (14). There is emerging evidence that direction changes of external forces directly influence *α*_IIb_*β*_3_’s activation. A normal tensile force of 40 pN is insufficient to induce unfolding in biomembrane force probe experiments (15), whereas tangential forces as low as 20 pN can readily unfold *α_IIb_β_3_* (16, 17). Nevertheless, whether this directional sensitivity of integrin *α*_IIb_*β*_3_ contributes to platelet activation under mechanical stimulation remains unclear.

Meanwhile, the coagulation process can be weakened by platelet spherocytosis. Spherical platelets, which result from genetic defects or drug treatments (18–20), exhibit reduced thrombus formation, while maintaining the responsiveness to chemical agonists. For instance, spherical platelets can still be activated by adenosine diphosphate (ADP), and their aggregation rate is comparable to that of normal platelets (21). Although the precise mechanism remains unclear, morphology appears to be a key factor. Spherical platelets tend to roll, whereas discoid platelets tend to slide, suggesting that reduced activation may arise from altered mechanical stimulation, particularly through integrin anisotropy–dependent responses to external force during motion.

**Fig. 1.**
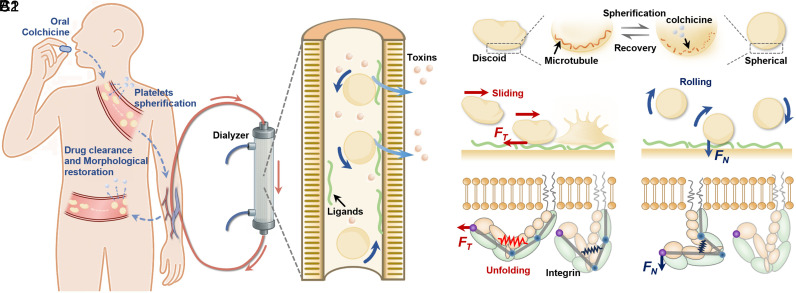
Schematics of the anticoagulation strategy based on platelet spherification and its underlying mechanism. (*A*) Application to extracorporeal life support, especially dialysis: colchicine induces transient spherification, which suppresses thrombus formation in the dialyzer while preserving responsiveness to soluble agonists. After dialysis, platelets recover their discoid shape. (*B*) Morphological changes suppress motion-dependent platelet activation. After colchicine treatment, platelets convert from a discoid to a spherical shape. Spherical platelets roll along the wall, whereas discoid platelets preferentially slide. The change is reversible after colchicine is metabolized. (*C*1) During sliding, a tangential force (*F_T_*) transmitted through the *α_IIb_β_3_*–ligand bond unfolds the integrin. (*C*2) During rolling, the dominant normal force (*F_N_*) pulls on *α_IIb_β_3_* without unfolding.

It is inspiring to consider that platelet spherification could serve as a potential option for anticoagulation strategy in extracorporeal systems, such as hemodialysis. In this work, we propose a feasible solution based on this concept and provide a detailed investigation of its underlying biomechanical mechanisms. Specifically, discoid platelets undergo spherification following treatment with colchicine, while their responsiveness to soluble agonists remains intact. These spherical platelets then circulate through the hemodialyzer, where coagulation induced by friction is effectively inhibited. After completing the extracorporeal treatment, the spherical platelets in the bloodstream revert to their original discoid shape (Fig. 1*A*). To connect this therapeutic potential with its mechanistic basis, we investigate how shape-dependent motions regulate platelet activation (Fig. 1*B*). The motion-dependent activation of platelets will be observed using an in vitro microfluidic model, and *α_IIb_β_3_* unfolding under sliding and rolling motions will be detected by atomic force microscopy (AFM). The biomechanical mechanism underlying *α_IIb_β_3_* unfolding under sliding and rolling motion conditions is elucidated through molecular dynamics (MD) simulations (Fig. 1*C*). These studies reveal that the anisotropic force sensitivity of integrins links spherical platelet rolling with reduced tangential stimulation and diminished activation. Finally, we demonstrate the application of platelet spherification in hemodialysis using low-dose colchicine. The in vitro results indicate that platelet spherification is an effective strategy to reduce coagulation risk in extracorporeal systems without increasing bleeding risk.

## Results

### Microfluidic Modeling of Platelet Spherification–Induced Anticoagulation.

**Fig. 2.**
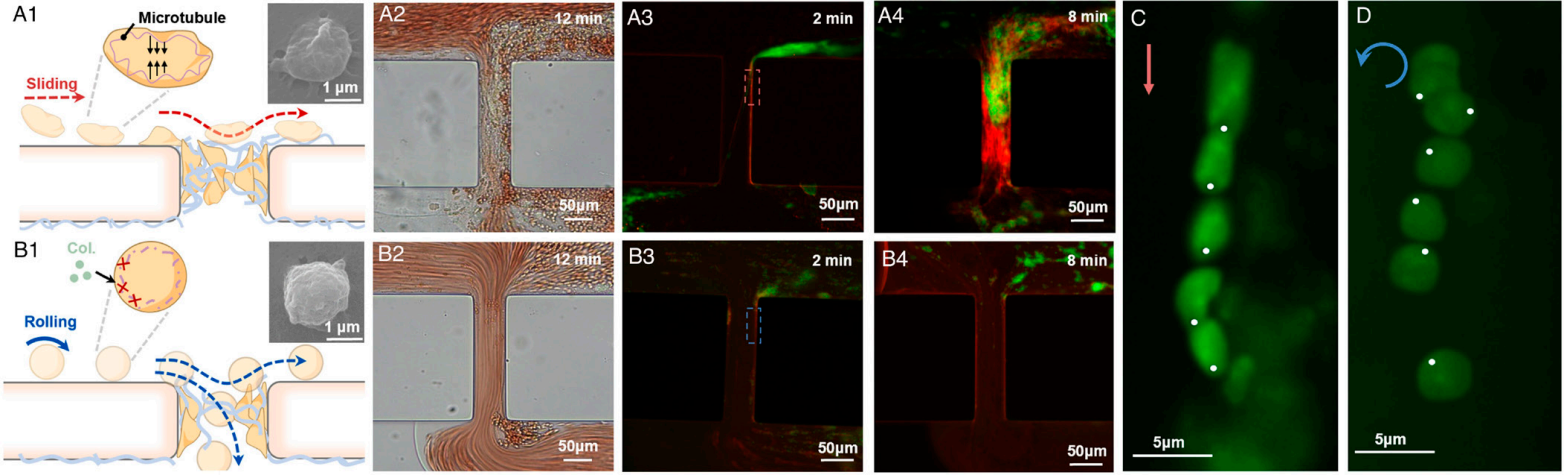
Thrombus tests in a microfluidic model with discoid and spherical platelets, respectively. (*A*1) Schematics of thrombus formation induced by discoid platelets. The inserted images are discoid platelets observed under SEM. (*A*2) Hemostasis testing in the control group was performed using microfluidic devices. Thrombus forms in the vertical channel, leading to complete flow blockage after 12 min. (*A*3 and *A*4) Fluorescent images of the thrombus in the vertical channel at the time of 2 and 8 min. (*B*1) Schematics of anticoagulation induced by spherical platelets. The inserted images are spherical platelets observed under SEM. (*B*2) Thrombus testing result of the experiment group performed in microfluidic devices. The blood is flowing and no channel occlusion occurred in 12 min. (*B*3 and *B*4) Fluorescent images of the blood flow in the vertical channel at the time of 2 and 8 min, and no thrombus was observed there. (*C*) Time-lapse composite image of a discoid platelet sliding along the channel surface on the right side of the image. Images were acquired at 1 s intervals. The white dot marks the contact point with the wall at the initial frame to trace the platelet trajectory. (*D*) Time-lapse composite image of a spherical platelet rolling anticlockwise along the channel surface on the right side of the image. The time-dependent variations in the displacement and rotation angle of the platelets in (*C* and *D*) are plotted in *SI Appendix,* Fig. S1 *H* and *I*. The movies of the hemostasis process in the microfluidic device and the motions of the platelets are provided in Movies S1–S4.

We develop a microfluidic chip (*SI Appendix,* Fig. S1*A*) to investigate the effect of platelet spherification on anticoagulation. Blood with spherical platelets was perfused in microfluidic channels, and the resulting state of blood flow in the channel was compared to that observed in blood with discoid platelets (Fig. 2 *A*1 and *B*1). The microfluidic model features an H-shape, with two horizontal channels for blood perfusion and buffer flushing, connected by a vertical channel simulating a vascular wound (*SI Appendix,* Fig. S1*B*). To simulate the intravascular environment, the buffer flushing channel and the vertical channel are coated with collagen to mimic the wound environment (*SI Appendix,* Fig. S1*C*), while the blood perfusion channel is coated with bovine serum albumin (BSA) to inhibit protein adsorption. Blood is perfused through the channel at a flow rate of 4.5 μL/min, resulting in a shear rate of 600 s^−1^, which is known to induce *α_IIb_β_3_*-dependent platelet activation in venous flow conditions. The buffer flushing channel is perfused with PBS buffer at a flow rate of 1 μL/min. The details on the platelet treatment and the results of spherification are provided in the *SI Appendix*.

After 12 min of perfusion, the vertical channel is occluded with thrombus in the control group where the untreated platelets were discoid, but remained unobstructed in the experiment group containing spherical platelets (Fig. 2 *A*2 and *B*2). In the control group, green-fluorescent platelets accumulated within the vertical channel alongside the developing red fibrin network (Fig. 2 *A*3 and *A*4). The channel was filled with green platelets and red fibrin after 8 min, and blood flow in the vertical channel ceased at 12 min (*SI Appendix,* Fig. S1 *D* and *E*).

Conversely, in the experimental group with platelet-spherification treatment, few platelets were observed within the vertical channel (Fig. 2 *B*3 and *B*4), and the channel remained unobstructed with the flow rate decreasing by only 32% after 20 min of blood perfusion (*SI Appendix,* Fig. S1 *D* and *E*). It indicates that platelet spherification avoids platelet activation and reduces the thrombus formation, which demonstrates the anticoagulation potential of spherical platelets.

The fraction of spherical platelets was set to 50% by mixing treated and untreated platelets while keeping the total platelet count constant. Under this condition, hemostasis was achieved after 12 min of blood perfusion. However, fibrinogen (red fluorescence) was markedly reduced and the resulting thrombus was looser. This indicates that although the remaining discoid platelets can still support hemostasis at 50% sphericity, the overall coagulation capacity and clot quality are compromised. The results are provided in *SI Appendix,* Fig. S1*G* and Movie S7.

Further observations reveal that the morphological change of the platelets alters the motion of the platelets. The platelet motions in the vertical channel were observed using an inverted microscope (DMi8, Leica Co.). Discoid platelets slide along the surface, gradually decelerating and eventually adhering to it (Fig. 2*C* and Movie S3); while spherical platelets roll over the surface, gradually accelerating and ultimately detaching (Fig. 2*D* and Movie S4), as shown in the plots of time-dependent variations in the displacement and rotation angle of the platelets in *SI Appendix,* Fig. S1 *H* and *I*, respectively. These results indicate that platelet morphology has limited impact on margin.

Platelet morphology can influence hemodynamic behavior, including margination and platelet–wall interactions. Although discoid and spherical platelets show different margination dynamics, spherical platelets still efficiently marginate toward the near-wall region and may even collide with the wall more frequently than discoid platelets (22–24). Thus, the reduced thrombosis observed here is unlikely to result from insufficient wall contact. Instead, the key difference appears to lie in motion style after wall contact: As shown in Figs. 1 and 2, discoid platelets tend to slide and undergo high-force activation, whereas spherical platelets tend to roll. The rod-sliding experiments described below further separate the effect of motion style from that of morphology by testing whether spherical platelets become activated when forced to slide. These findings indicate that motion style and the associated tangential force, rather than shape-dependent margination alone, dominate platelet activation in our model. Consistent with this, spherical platelets showed a 3.5-fold higher probability of rolling than discoid platelets (*SI Appendix*, Fig. S1*F*), suggesting a potential link between spherification-induced rolling and anticoagulation.

### Activation Capacity of Spherical Platelets.

To investigate the anticoagulation mechanism of platelet spherification, it is essential to evaluate the activation capacity of spherical platelets, including their responses to biochemical agonists, external forces, and activation signaling pathways, and their performances are analyzed and compared with those of the normal discoid platelets.

**Fig. 3.**
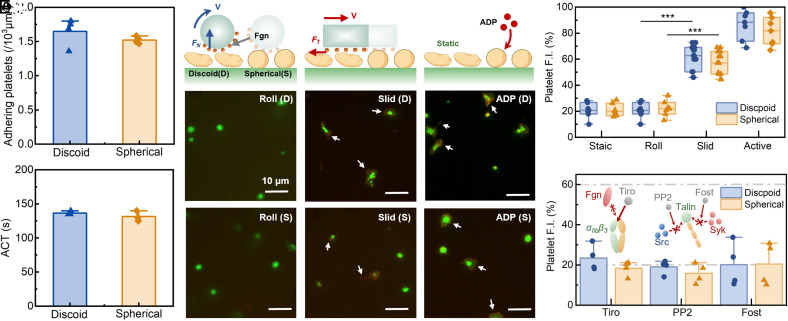
Experimental evaluation of platelet activation capacity after spherification. (*A*) Number of adhered platelets under static incubation with discoid and spherical platelets, respectively. (*B*) Activated clotting time (ACT) measured in discoid and spherical platelets. (*C*) Schematic and representative fluorescence images of discoid and spherical platelets stimulated by a rectangular rod (sliding), a round rod (rolling), or ADP agonist. The white arrows indicate regions of pseudopod formation and spreading, which also exhibit strong yellow fluorescence. (*D*) Fluorescence intensity (F.I.) of discoid and spherical platelets under static conditions, sliding, rolling, or ADP stimulation. (*E*) Fluorescence intensity of platelets under sliding stimulation with pathway inhibition: integrin–fibrinogen binding blocked by tirofiban (Tiro), Src inhibition by PP2, and Syk inhibition by fostamatinib (Fost).

Platelet adhesion assays and Activated coagulation time (ACT) detection were first performed. It is found that there is no significant difference on the number of adhering platelets and the ACT between discoid and spherical platelets (Fig. 3 *A* and *B* and *SI Appendix,* Fig. S2 *C* and *D*), indicating that platelet spherification does not compromise its adhesive capacity or responsiveness to biochemical agonists.

Next, the activation capacity of spherical platelets under external forces is examined. Rod-sliding experiments were performed using rods of different geometries to control the orientation of applied forces, which provide a macroscopic manipulation platform to evaluate whether force-direction-dependent activation persists at larger scales. Platelets were anchored to glass surfaces through a dopamine coating preventing premature adhesion-induced activation. A round rod 8 mm in diameter rolled continuously along the glass surface to apply rolling frictional force on platelets (Fig. 3*C*1), while a rectangular rod 10 × 5 mm in size slid along the surface to exert sliding frictional force on platelets (Fig. 3*C*2). Both rods were coated with fibrinogen to provide ligand binding sites. Platelets were stained with the green fluorescent dye DiOC6 (D273, ThermoFisher), while activated platelets were labeled with CD62P-PE (12-0626-82, Thermo Fisher), which generated yellow fluorescence. Experiments were performed for 10 min, static and ADP-treated platelets were served as negative and positive controls, respectively (Fig. 3*C*3). Under sliding motion condition, both discoid and spherical platelets exhibited strong yellow fluorescence, accompanied by pseudopod formation and spreading (Fig. 3 *C*5 and *C*8). In contrast, under rolling motion, only weak fluorescence appeared in both platelet types (Fig. 3 *C*4 and *C*7). No significant differences were observed between spherical and discoid platelets in any of the four assays (Fig. 3*D*), which confirms that the activation capacity of spherical platelets under external force remains undamaged and is the same as that of discoid platelets.

Additionally, the activation signaling pathway of spherical platelets under external forces was examined. In this experiment, the external force on platelets is generated from the interaction between integrin *α*_IIb_*β*_3_ and surface-immobilized ligand fibrinogen. This force is transmitted through an outside-in signaling pathway, which connects *α*_IIb_*β*_3_ to the actin cytoskeleton and promotes the extended-open (EO) conformation of *α*_IIb_*β*_3_ (25). This process begins with Talin binding to the β-subunit tail, which anchors integrins to the cytoskeleton. Src is then recruited to phosphorylate sites on the β-subunit, reinforcing cytoskeletal anchoring. This reinforcement maintains the EO conformation of *α*_IIb_*β*_3_. Subsequently, Syk is activated, followed by downstream signaling through FAK, PLCγ2, and ADAP, ultimately leading to platelet activation (26). To verify the integrity of this activation pathway of spherical platelets, three key nodes were inhibited: integrin *α*_IIb_*β*_3_–ligand binding (blocked by tirofiban, ST9610, Solarbio), Src (inhibited by PP2, T6266, TargetMol), and Syk (inhibited by fostamatinib, T0458, TargetMol). The three inhibitors were applied separately, and each kind of inhibitor caused suppressed platelet activation under sliding mechanical stimulation in both platelet types; the yellow fluorescence of the spherical platelets reduced to the level of static control (Fig. 3*E*). These results confirm that spherical platelets retain the same outside-in signaling pathway under external forces. As an extension, we also varied the sliding fraction by changing the incline of the glass. As the sliding fraction increased from 0 to 100%, yellow fluorescence rose from 20 to 60% (*SI Appendix,* Fig. S3). Platelet activation is modulated by the proportion of tangential force applied during motion.

According to the activation measurements, platelet spherification does not compromise the intrinsic activation capacity of platelets. The exhibited anticoagulation effect of spherical platelets in the microfluidic experiments is attributed to the altered motion of platelets from sliding to rolling. The rolling motion suppresses platelet activation, and the underlying mechanism is investigated as follows.

### AFM Detects the Motion-Dependent Unfolding of *α_IIb_β_3_*.

**Fig. 4.**
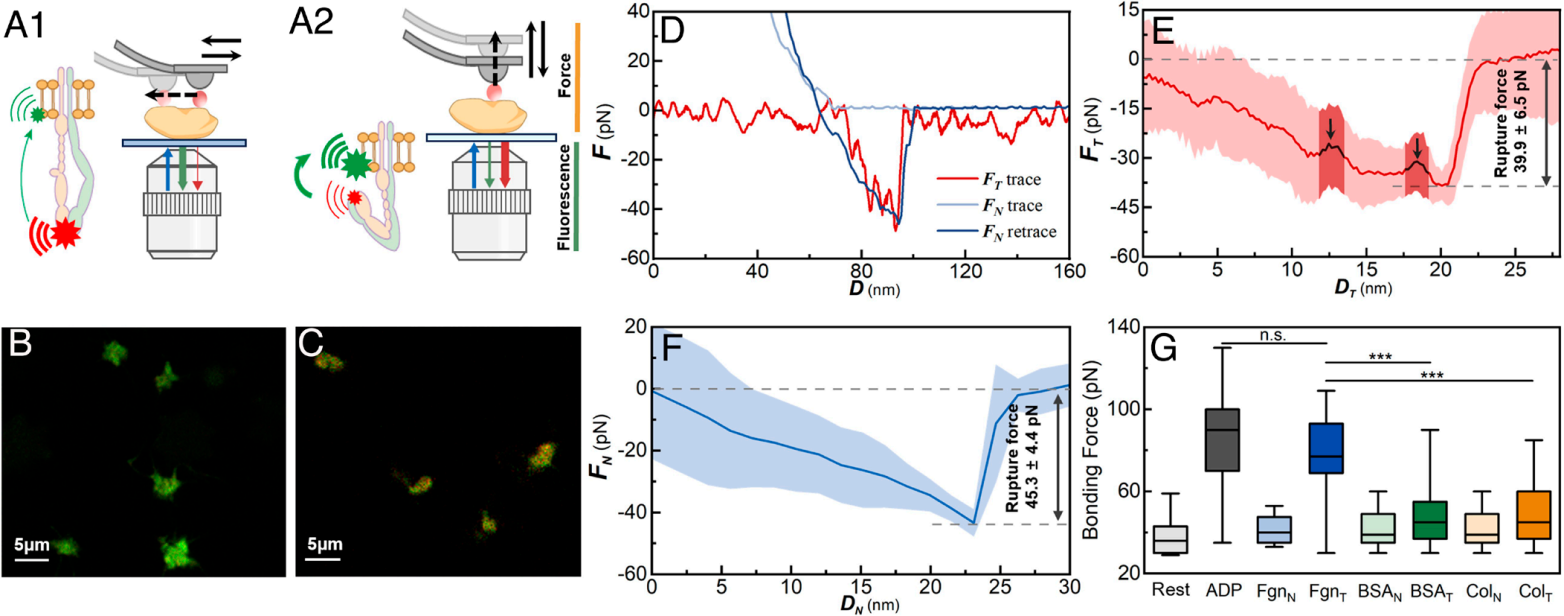
Integrin unfolding under sliding and rolling conditions detected by AFM. (*A*1) In contact mode, tangential force is applied to platelets. The blue arrow represents incident light, the green arrow represents reflected fluorescence from the integrin head, and the red arrow represents reflected fluorescence from the cell membrane. (*A*2) Tapping mode, normal force is applied. The red fluorescence is enhanced by the FRET effect. (*B*) Platelets present green fluorescence in contact mode, indicating *α_IIb_β_3_* is unfolded with a weak FRET effect. (*C*) Platelets present red and green fluorescence in tapping mode, indicating *α_IIb_β_3_* is still folded with a strong FRET effect. (*D*) The representative force–displacement curve of inactivated *α_IIb_β_3_* under tangential (red) and normal (blue) force. (*E*) The tangential force–displacement curves on inactivated *α_IIb_β_3_* using contact mode. The red line is the average force of 40 tests, and the shadow area represents the SD. (*F*) The normal force–displacement curves on inactivated *α_IIb_β_3_* using tapping mode. The blue line is the averaged result of 40 tests, and the shadow area represents the SD. (*G*) Bonding force between *α_IIb_β_3_* and RGDs after *α_IIb_β_3_* was exposed to fibrinogen (Fgn), BSA, and Collagen (Col). The subscript “N” represents results stimulated by the normal force in the tapping mode. The subscript “T” represents results stimulated by tangential force in the contact mode.

To mimic the friction forces on the platelets rolling and sliding along the wall, we applied precise loads to single platelets with AFM. The contact mode of AFM was employed to mimic a physiological scenario wherein a discoid platelet slides on the ligand. An AFM probe coated with RGD peptides (91037-65-9, ChinaPeptides Co.) was used to reciprocally slide across a platelet adhered to a surface using dopamine (P512501, HX-R Co.) (Fig. 4*A*1). The sliding speed of the probe is 0.8 μm/s according to the platelet moving speed on the vein’s surface (27). The tapping mode of AFM was used to model the rolling of a spherical platelet on the ligand. The probe approaches platelets normally and then retracts after contacting the platelet. This approach-retract motion is repeated to mimic the periodic detachment of *α_IIb_β_3_* from ligands during the rolling motion (Fig. 4*A*2). The normal loading speed is 0.8 μm/s according to the speed of single integrin–ligand dissociation (28). The scanning speed is 0.8 μm/s, the same as that in contact mode. In the experiment, the applied speed of 0.8 μm/s is consistent with the intrinsic kinetics of integrin activation reported in the literature. For example, a conformational shift of ~15 nm occurring at 0.8 μm/s takes approximately 18.75 ms. This is well within the physiological switching time (~50 ms) of integrin αLβ2 unbending measured via biomembrane force probe (BFP) (29). The pulling rate (0.8 μm/s) also aligns with standard AFM single-molecule/cell force spectroscopy, which typically operates in the 0.5 to 1.0 μm/s range to resolve specific integrin–ligand interactions [α_2_β_1_ at 0.9 μm/s (30) and α_5_β_1_ at 1.0 μm/s (31)]. More details on the settings of AFM are seen in the *SI Appendix*.

Simultaneously, the unfolding of *α*_IIb_*β*_3_ is detected by the fluorescence response after the AFM experiment. The platelet membrane is labeled in green fluorescence using DiO (C1036, Beyotime Co.), and the head of *α*_IIb_*β*_3_ is tagged with red fluorescence by Rhodamine B-RGDS (ChinaPeptides Co.). The head-to-membrane Förster resonance energy transfer (FRET) configuration used here is a well-established approach to report integrin conformational activation (32, 33). We validated the labeling efficiency and specificity of both FRET partners: The lipophilic membrane tracer (DiO) showed peripheral localization with the corresponding Fluorescence Lifetime Imaging Microscopy (FLIM) signature, and the integrin-head probe (Rhodamine B-RGDS) was enriched on the platelet surface. Specific binding to αIIbβ3 was further confirmed by a tirofiban competition assay, which markedly reduced the Rhodamine B-RGDS signal. The details on the labeling validation and associated FLIM/phasor analyses are provided in the *SI Appendix,* Fig. S4).

FRET is most sensitive to donor–acceptor separations within the nanometer range; it has been widely used as a semiquantitative reporter of large-scale integrin conformational rearrangements. Existing research has shown that the distance between the resting membrane and the integrin head is 3.5 to 4 nm, and the distance after activation is 19 nm. The distance difference is often discussed as ~15 nm for extension. Because FRET efficiency decays steeply with distance, it is most sensitive when donor–acceptor separations lie roughly in the ~1 to 10 nm range. Such an extension magnitude (15 nm) will significantly reduce the FRET effect.

When *α*_IIb_*β*_3_ unfolds, its head moves away from the membrane which eliminates the Förster resonance energy transfer (FRET) effect (34) and prevents the green fluorescence from enhancing the red fluorescence on the membrane (Fig. 4*A*1). When *α*_IIb_*β*_3_ is in the bent-closed conformation, its head is close to the membrane, and the red fluorescence will be enhanced by the green (Fig. 4*A*2). In the experiment, red fluorescence intensity remained low under the contact mode (Fig. 4*B*).

The FLIM–FRET measurements were included which does not require absolute fluorophore concentrations, because fluorescence lifetime is independent of intensity fluctuations and concentration differences (35), and it also enables mapping of FRET-active regions on the cell surface. As shown in *SI Appendix,* Fig. S5, regions exhibiting detectable FRET consistently display high FRET values, indicating the presence of the bent-closed (BC) integrin state in these areas. However, the spatial extent of these high-FRET regions differs markedly across conditions. Resting platelets and platelets subjected to normal mechanical stimulation maintained large surface areas with high FRET, with an apparent FRET efficiency of ~60%. In contrast, activated platelets showed pronounced spreading, with high-FRET signals scattering in the central region and the apparent FRET efficiency decreasing to ~20%. The conformational landscape can be robustly mapped through the apparent FRET efficiency. αIIbβ3 molecules are usually estimated at ~80,000 copies per cell. In the experiment, the αIIbβ3 molecules in resting status exhibited a high FRET efficiency of ~60% across the entire cell surface. This indicates that the vast majority of labeled receptors exist in the bent-closed (BC) conformation, maintaining a uniform spatial distribution. The application of tangential forces by the AFM tip triggered a dramatic shift in integrin conformation. In the spreading regions, the apparent FRET efficiency decreased to ~20%. Therefore, we estimate that 70% of the surface integrins in this region, approximately 56,000 molecules, shifted to the active conformation.

The FRET efficiency of 63% is obtained (*SI Appendix,* Fig. S5*D*). The Förster radius was calculated as R_0_ = 0.02108(κ^2^ Φ_D_ n^−4^ J)^1/6^ = 5.25 nm (32), where the orientation factor κ^2^ was assumed to be 2/3, the refractive index n of PBS is 1.33, and the quantum yield of DiO Φ_D_ was set to 0.4. The spectral overlap integral J(λ)was calculated from the area-normalized emission spectrum of DiO and the absorption (excitation) spectrum of Rhodamine B. Based on these parameters, the inferred head-to-membrane distance in the bent-closed (BC) state is 4.8 nm, which is consistent with previous reports (36). In contrast, tapping mode exhibited an enhanced red fluorescence on platelets (Fig. 4*C*). The FRET-based analysis of integrin conformation provides experimental evidence that external force can directly unfold integrin α_IIb_β_3_. These results also validate that tangential friction forces from sliding, but not normal forces from rolling, induce α_IIb_β_3_ unfolding.

To further characterize the specific unfolding mechanics of integrins, we performed AFM force-distance curve measurements. In contact mode, the tangential friction force *F*_T_ is detected during the sliding motions, as presented in the force–displacement curves (Fig. 4*D*). In each test, the force experienced a sudden jump, characterized by a slow decrease to −39.9pN and then a rapid increase to 0 pN. The rapid increase in force is attributed to α_IIb_β_3_–RGD binding, which induces lateral twisting of the probe along the surface scanning direction. Until the RGDs on the probe dissociate from *α_IIb_β_3_*, the probe cantilever returns to its resting position, and the jump occurs. These force jumps are often observed in AFM experiments during integrin–ligand detachment, and the measured force, 39.9 ± 6.5 pN, is also consistent with the reported binding force range of 30 to 50 pN for inactivated *α_IIb_β_3_* interacting with RGDs (37). Significantly, two distinct prerupture peaks were detected before the sudden jump (Fig. 4*E*). With amplitudes of 2.2 and 2.15 SD of the signal (*SI Appendix,* Fig. S6*B*), both peaks exceeded the statistical significance threshold of 1.96 SD of the noise (38, 39), which distinguished them from background noise. In the AFM force–displacement curves, small peaks appeared before the final rupture event. These prerupture peaks are consistent with stepwise dissociation between integrin subunits and the peak amplitudes are ~6 pN, in line with reported strengths (40). To confirm that the peaks are caused by the unfolding of *α_IIb_β_3_*, the force on activated *α_IIb_β_3_* is also detected. The activation of *α_IIb_β_3_* is achieved by introducing an ADP solution before the experiments. As predicted, the force–displacement curve for activated *α_IIb_β_3_* exhibited no peaks before the jump (*SI Appendix,* Fig. S6*C*). Therefore, the observed peaks validate the *α_IIb_β_3_* unfolding induced by sliding motion.

In tapping mode, the jump is also observed in the force–displacement curves with a measured amplitude of 45.3 ± 4.4 pN, which is also consistent with the same binding force range of 30 to 50 pN for inactivated *α_IIb_β_3_* interacting with RGDs (37) (Fig. 4 *D* and *F*). However, there is no prerupture peak on the curve before the jump. The absence of prerupture peaks suggests that there is no domain separation within the *α_IIb_β_3_* domains, and the integrin maintained its original bent-closed state until the *α_IIb_β_3_*–RGD bond was dissociated. This finding validates that the normal forces in the rolling motion of platelets are insufficient to induce *α_IIb_β_3_* unfolding.

It should be noted that the unfolding of *α_IIb_β_3_* only means that *α_IIb_β_3_* enter an extended-close state, but there is still a step to the extended-open state, which features the activation of the integrin. The experiment results above are on the platelets with the inhibitors to stop the transition from the extended-close state to the extended-open state, so the unfolding of integrin *α_IIb_β_3_* can be observed without the interference from previously activated integrins.

To investigate *α_IIb_β_3_* activation in the extended-open state under rolling and sliding motions, AFM experiments were performed on the platelets without inhibitors. Fibrinogen-coated probes were continuously scanned over platelets in contact mode and tapping mode. After 6 min of scanning, the probes were replaced by RGD-coated probes to measure the binding force between *α_IIb_β_3_* and its RGD ligand. This replacement was performed to prevent contamination or blunting of the probe surface after scanning and to ensure the reliability and consistency of force measurements. The binding forces observed during the two distinct motions were compared against the binding force exhibited by resting platelets, where *α_IIb_β_3_* adopts a bent-closed conformation, and that of ADP-treated platelets, where *α_IIb_β_3_* is in its extended-open state. The RGD-binding force observed during sliding was comparable to that in ADP-activated platelets and higher than that observed during rolling or in resting platelets (Fig. 4*G*). This result indicates that tangential forces from the sliding motion can effectively activate *α_IIb_β_3_*.

Meanwhile, BSA and collagen were employed to simulate the intact and injured vascular surfaces. To detect the interactions of *α_IIb_β_3_* with these proteins, probes were coated with BSA or collagen. However, neither sliding motion nor rolling motion causes the increase of binding force on the coated probes (Fig. 4*G*). At the same time, vWF is absent and no high-shear flow is applied, making GPIb-dependent mechanotransduction unlikely. Although RGD can bind α_V_β_3_, its platelet expression is low and is not preferentially engaged under our coating conditions. It confirms the specificity of *α_IIb_β_3_* activation by fibrinogen.

These AFM experiments directly demonstrate that the unfolding of integrin *α_IIb_β_3_* depends on the direction of applied mechanical force. Tangential forces generated during sliding motion induce stepwise unfolding of *α_IIb_β_3_*, while normal forces produced during rolling fail to do so. This directional mechanosensitivity explains why spherical platelets, which predominantly roll along surfaces, exhibit reduced mechanical activation and lower thrombotic potential.

### MD Simulates *α_IIb_β_3_* Unfolding Pathways.

**Fig. 5.**
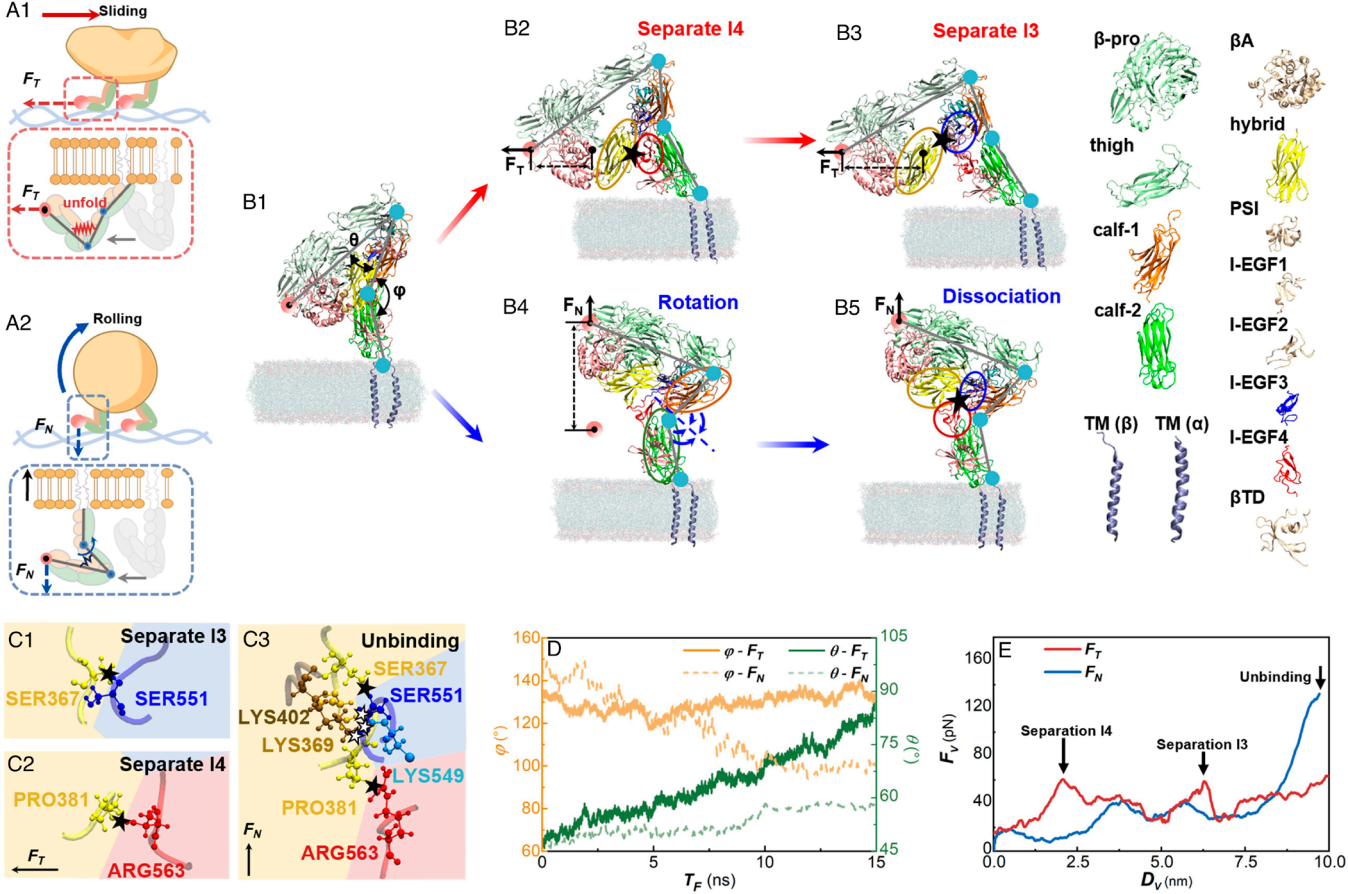
Simulated unfolding process of *α_IIb_β_3_* using SMD. (*A*1 and *A*2) Schematics of unfolding of *α_IIb_β_3_* during sliding of discoid platelets and rolling of spherical platelets. (*B*1) The structure of *α_IIb_β_3_* with α subunit and β subunit. Calf-1 is marked in orange, and Calf-2 is marked in green, which are located on the α subunit. Hybrid (yellow) and I-EGF domains are located on the β subunit. I-EGF domains are a class of domains containing characteristic EGF-like (Epidermal Growth Factor) folds including I-EGF3 (blue) and I-EGF4 (red). The red arrows indicate the integrin conformational changes induced by tangential pulling, whereas the blue arrows indicate the conformational changes induced by normal pulling. (*B*2) The separation of Hybrid and I-EGF4 in stage “Separation I4” and (*B*3) the separation of Hybrid and I-EGF3 in stage “Separation I3.” (*B*4) The head of the integrin rotates in stage “Rotation” under normal force, and the normal traction force cannot separate the binding between H-I4 and H-I3, and (*B*5) the ligand is detached from *α_IIb_β_3_* in stage “Dissociation.” (*C*1 and *C*2) Hydrogen bonds between Hybrid and I-EGF3 (H-I3) and between Hybrid and I-EGF4 (H-I4) under sliding condition, hydrogen bonds are indicated by solid stars. (*C*3) Hydrogen bonds between H-I4 and between H-I3 under rolling condition, new hydrogen bonds are marked by hollow stars. (*D*) The variation of *θ* and *φ* during the sliding and rolling motion conditions. (*E*) The variation of force on the traction point in the constant-velocity SMD. The process of *α_IIb_β_3_* conformational changes under tangential and normal force is provided in Movies S5 and S6.

An all-atom model of *α_IIb_β_3_* was developed using GROMACS (41) to investigate the biomechanical mechanisms underlying integrin unfolding under different motion conditions. The conformational responses of *α_IIb_β_3_* under sliding-like and rolling-like loading are simulated using MD. The simulations show distinct unfolding pathways. During sliding, platelets need to overcome the tangential friction force *F*_T_ (42), generated by *α_IIb_β_3_* binding to surface ligands. This force will induce *α_IIb_β_3_* unfolding and a subsequent conformational change (Fig. 5*A*1). During rolling, detachment of *α_IIb_β_3_* from the ligand generates a normal pulling force *F*_N_ (43), which will induce a conformational change distinct from that caused by tangential forces (Fig. 5*A*2).

The type of MD simulation that applies external force is called Steered Molecular Dynamics (SMD). During the SMD simulation, the tangential and normal forces of 70 pN are applied at the ligand tirofiban in the head of the β subunit of *α_IIb_β_3_* for stable traction (44) as shown by the pink circle (Fig. 5*B*1). Conformational changes of the integrin *α_IIb_β_3_* induced by external forces are quantified by two angles on its α subunit: *θ*, the angle formed between the Thigh domain and the Calf-1 domain, and *φ φ*, the angle formed between the Calf-1 and the Calf-2 (Fig. 5*B*1).

During sliding, the unfolding of *α_IIb_β_3_* experiences two typical conformation changes, defined as “Separation I4” and “Separation I3.” The stage of Separation I4 happens first, where the Hybrid is separated from I-EGF4 in the β subunit, as shown by the yellow and the red ovals (Fig. 5*B*2). The separation needs to overcome one hydrogen bond, PRO381-ARG563 (PRO: Proline, ARG: Arginine) between the two domains (Fig. 5*C*2) and the separated hydrogen bond is marked with a solid star. In the following stage Separation I3, Hybrid is separated from I-EGF3 in β subunit (Fig. 5*B*3). The separation needs to overcome another hydrogen bond, SER367-SER551 (SER: Serine) (Fig. 5*C*1). This separation causes angle *θ* to increase from 45° to 75°, while angle *φ* is almost unchanged (Fig. 5*D*). Correspondingly, the interaction energies *E_S_* between the Hybrid domain and the I-EGF3 or I-EGF4 domains decrease from 80 to 0 kJ/mol (*SI Appendix,* Fig. S7*E*). The tangential force disrupts these interactions, thereby separating the integrin head from the lower leg region and leads to the unfolding of *α_IIb_β_3_*. The two stages explain the two prerupture peaks observed in AFM contact-mode results.

During rolling, the conformational changes of *α_IIb_β_3_* experience two stages: Rotation and Dissociation. In the Rotation stage, α_IIb_β_3_ is observed to rotate around the junction between the calf-1 and calf-2, resulting in the decrease of *φ* from 140° to 100°, while *θ* does not change because Hybrid, I-EGF4, and I-EGF3 are still bonded together without separation (Fig. 5*B*4). The rotation will be stopped when Calf-1 contacts Calf-2, and then the Dissociation stage begins (Fig. 5*B*5). Due to the contact of Calf-1 to Calf-2, more hydrogen bonds are formed: LYS549 forms two additional hydrogen bonds with LYS402 and forms one additional hydrogen bond with SER369, as shown by hollow stars (Fig. 5*C*3). Under these conditions, the integrin remained folded throughout the simulation before the final dissociation of the integrin from the ligand. This result agrees with AFM tapping-mode results.

The forces during the separation of hydrogen bond in stages of Separation I3 and I4 are acquired as 60 pN and 58 pN under a constant velocity pulling condition. Details of the pulling parameters are provided in the *SI Appendix*. However, more than 140 pN is required to dissociate the five hydrogen bonds at the Dissociation stage (Fig. 5*E*). SMD is constrained by computational cost, and the pulling/loading rates used in simulations are typically far higher than in AFM, and therefore absolute force magnitudes are not expected to match one-to-one. In the SMD results, the calculated forces are approximately twofold higher than the experimental values, which is plausible given the ~10^6-fold higher effective loading rate implemented in the computational model (45). Since the dissociation force between *α_IIb_β_3_* and RGD is 70 pN (27), which is smaller than the dissociation force of the hydrogen bonds, the normal pulling force will cause *α_IIb_β_3_* to detach from ligands before the hydrogen bonds are dissociated. Therefore, the integrin *α_IIb_β_3_* will not be unfolded by the normal force in rolling condition. Molecular dynamics simulations revealed the phenomenon: Force alone can unfold integrin *α_IIb_β_3_*, and due to *α_IIb_β_3_*’s asymmetric conformation, the unfolding exhibits anisotropic resistance depending on force direction.

The cytoplasmic-tail conformations were compared before and after SMD (*SI Appendix,* Fig. S7*B*). The tails exhibited only minor conformational changes and did not undergo appreciable extension under either pulling mode.

### In Vitro Hemodialysis Experiments.

**Fig. 6.**
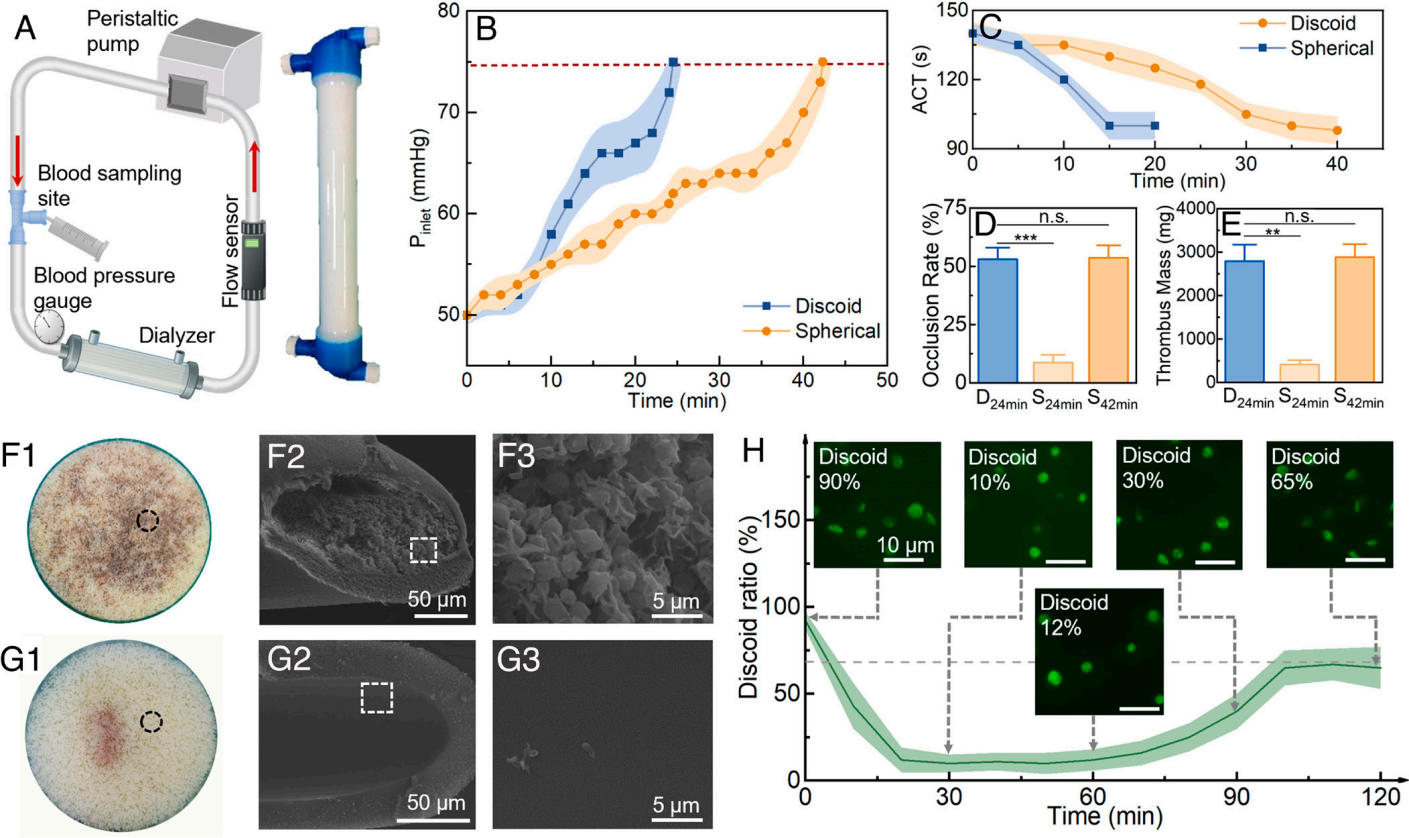
In vitro dialysis experiments using platelet spherification for anticoagulation. (*A*) Schematic of the in vitro hemodialysis setup, along with photographs of the dialyzer. (*B*) Inlet pressure during dialysis experiments. The starting value was 50 mmHg, and experiments were terminated once the inlet pressure rose by more than 25 mmHg. (*C*) Changes in activated clotting time (ACT) during the in vitro hemodialysis experiments. (*D*) Occlusion ratio of dialyzer fibers. D represents discoid platelets, S represents spherical platelets, and subscripts indicate the circulation duration. (*E*) Thrombus mass within the dialyzer after experiments. (*F*) Dialyzer from the untreated control group (D_24min_, D represents discoid platelets, 24 min indicates the circulation time) showing extensive fiber occlusion at the cross-section. SEM images reveal dense thrombus formation along the inner fiber walls. (*G*) Dialyzer from the second spherification group (S_24min_) showing limited fiber occlusion at the cross-section. SEM images display the clean inner fiber walls with minimal adhesion of blood cells. (*H*) Temporal changes in the discoid ratio of platelets during the second spherification group (S_24min_), with representative fluorescence images at four characteristic time points. From 0 to 30 min, platelet spherification phase induced by colchicine; 30 to 60 min, blood circulation phase; 60 to 120 min, recovery phase when platelets reverted to a discoid shape.

To validate the anticoagulation effect of platelet spherification in extracorporeal life support, we selected hemodialysis as an experimental model. Hemodialysis employs hollow-fiber membranes that mimic renal function, allowing toxins and water to cross into dialysate. A typical dialyzer contains approximately 10,000 hollow fibers, each with a diameter of 200 μm and a length of 25 cm. In the narrow fibers, platelets migrate along the inner surface, and blood flow through such narrow fibrous tubes typically increases the risk of coagulation. An in vitro hemodialysis circulation model was constructed using FX5 dialyzers (Fresenius Co.) with the membrane area of 1 m^2^ (Fig. 6*A*). The extracorporeal circuit consisted of a blood loop and a simplified dialysate loop. The blood loop was driven by a peristaltic pump (YZ1515X, Runze Fluidics Co.) and monitored by a flow sensor (FD-XS20, Keyence Co.) to maintain a flow rate of 100 mL/min. A blood pressure gauge (2011F243-32, Yuwell Co.) was placed at the dialyzer inlet to monitor pressure changes, and a three-way valve was incorporated for blood sampling during circulation. The dialysate loop was driven by a peristaltic pump to maintain a constant flow of 200 mL/min of physiological saline. Meanwhile, colchicine was used to induce platelet spherification in these experiments. Colchicine, usually indicated for the treatment of gout, works by binding to tubulin, thereby inhibiting microtubule polymerization and altering cellular morphology. Clinical evidence demonstrates that colchicine is well tolerated by dialysis patients without evident toxicity (46). Sterile porcine blood was circulated through the blood loop. The control group’s blood is untreated, whereas the experimental group was treated with colchicine (20 nM) to induce platelet spherification.

The colchicine concentration (20 nM) was selected to approximate clinically relevant peak systemic exposure. Pharmacokinetic measurements in healthy volunteers indicate that a standard low-dose regimen yields a peak plasma concentration (C_max_) of ~6.19 ng/mL, corresponding to ~15.5 nM, placing our in vitro dose within the same order of magnitude as therapeutic exposure (47). Colchicine is often used as an oral prescription medication, but direct incubation of whole blood or platelet-rich plasma (PRP) with colchicine is a well-established methodological approach for investigating microtubule-dependent platelet functions (48). This method allows for the precise characterization of colchicine’s direct inhibitory effect on tubulin polymerization without the interference of systemic metabolic clearance.

During circulation, blood samples were collected every 5 min for activated clotting time (ACT) measurements. The experiment was terminated at the clotting time once the dialyzer inlet pressure increased by 50%, a threshold of significant clot formation (49). The termination point was recorded as the clotting time. Each experiment was independently repeated three times.

Using whole blood introduces blood cells other than platelets; however, under the nanomolar, short-exposure conditions used here, off-target effects on coagulation are expected to be minimal. Mature erythrocytes lack microtubules and rely mainly on the spectrin–actin membrane skeleton, so a direct mechanical effect of colchicine on RBC rheology is unlikely (50). In leukocytes, nanomolar colchicine primarily exerts anti-inflammatory actions (reduced chemotaxis/inflammasome signaling) rather than directly altering the core coagulation cascade (51). Meanwhile, our sterile, short-duration dialysis circuit lacks inflammatory agonists, making such contributions likely minor. Consistently, clinical data show no measurable changes in standard coagulation markers (PT, APTT, D-dimer, thrombin generation) with colchicine (52). Therefore, the reduced thrombosis arises primarily from platelet spherification and the associated change in mechanical activation mode.

In the experiments, the inlet pressure of the dialyzer in the control group increased more rapidly than that in the experimental group (Fig. 6*B*). The pressure of the control group reached 50% threshold at 24 min, and the clotting time was set to be 24 min (D_24min_). At this time point, the inlet pressure in the spherification group was only 61 mmHg, corresponding to a 22% rise from the starting pressure, which remained far below the 50% threshold (Fig. 6*B*). The clotting time of the experimental group was 42 min (S_42min_), representing a 75% prolongation. The measured ACT value declined from 140 s to 100 s after 15 min of circulation in D_24min_, while the value remained higher at 125 s in S_42min_ (Fig. 6*C*), indicating that platelet spherification significantly suppresses coagulation within the dialyzer and extends the duration of heparin-free dialysis.

After 24 min, dialyzers from both the D_24min_ and S_42min_ groups were assessed to determine the fiber occlusion ratio and the thrombus mass (Fig. 6 *D* and *E*). The D_24min_ group exhibits an occlusion ratio of 53% and a thrombus mass of 2,800 mg, whereas S_42min_ demonstrates a 54% occlusion ratio and a thrombus mass of 2,900 mg. To compare thrombus formation at equal circulation time, the second experimental group was terminated after 24 min (S_24min_), matching the clotting time of the D_24min_ group. The S_24min_ group showed 9% occlusion and a thrombus mass of 410 mg (Fig. 6 *D* and *E*), both markedly lower than those in the D_24min_ group.

In the D_24min_ group, the fibers blocked by thrombus form a brown area on the cross-section of the dialyzer (Fig. 6*F*1). Under scanning electron microscopy (SEM), the fiber was filled with a lot of blood cells and fibrin bundles (Fig. 6 *F*2 and *F*3). In contrast, only a small brown area was formed in the center of the cross-section of the dialyzers in the S_24min_ group (Fig. 6*G*1). A few platelets were found on the inner surface of the fiber (Fig. 6 *G*2 and *G*3). Therefore, platelet spherification demonstrates efficacy in suppressing platelet activation, which subsequently reduces thrombus formation during hemodialysis.

To prevent underestimation, our experimental circuit was equipped with a venous canister downstream of the hemofilter to capture any embolic debris or unstable thrombi dislodged during both the procedure and the final flush. We have quantified the thrombus mass collected in the venous canister and included this in our total assessment (*SI Appendix,* Fig. S8). The thrombus volume captured in the canister was significantly lower in the spherical platelet group (S_24min_) compared to the discoid group (D_24min_). While the collected thrombus volume in the S_42min_ group increased relative to S_24min_, it remained substantially lower than the D_24min_ control. These results demonstrate that the reduction in thrombus mass observed with spherical platelets is not due to increased embolization (wash-out), but rather a genuine decrease in overall thrombus formation.

Moreover, spherical platelets can regain their native discoid morphology once the colchicine is cleared. Using an inverted microscope, we continuously monitored freely suspended platelets at a fixed field of view and observed a gradual transition from spherical to discoid morphology over time. The supporting movie is added in Movie S8. Before the experiment, the morphology changes of the colchicine-treated platelet were observed with DiOC6 (Fig. 6*H*). During the initial 30 min of colchicine incubation, the proportion of discoid platelets rapidly decreased from 90 to 10% and remained unchanged in the next 30 to 60 min, confirming that colchicine effectively induced stable spherification. After the experiments, platelets were enriched, resuspended in PBS to remove colchicine, and incubated at 37 °C for 60 min. After drug removal (60 to 120 min), the spherical platelets gradually reverted toward the discoid shape with the proportion of 65% at 120 min. This morphological recovery was attributed to microtubule reassembly. This reversible change in platelet morphology indicates that the colchicine-induced platelet spherification method also allows platelets to resume their normal morphology after dialysis, thereby minimizing bleeding risk.

Microfluidic model and dialyzer experiment address platelet spherification at different spatial scales. The microfluidic assay (Fig. 2) resolves morphology-dependent platelet rolling/sliding dynamics and the resulting early thrombus formation at the microscale, whereas the dialyzer experiment (Fig. 6) serves as a device-scale validation under clinically relevant flow and pressure conditions, thereby linking the single-cell mechanism to blood-contacting device performance.

## Discussion

Spherical platelets suppress clot formation in extracorporeal circuits while their activation potential remains intact. Shape changes from discoid to spherical alter their motion preference from sliding to rolling, and thereby the direction of wall-imposed forces from tangential to normal. Tangential force from sliding promotes platelet activation, while normal force from rolling suppresses it, which is the basic principle underlying the motion-dependent anticoagulation method of platelet spherification. AFM and MD results reveal that force direction affects *α_IIb_β_3_* unfolding and the consequent platelet activation. The spherification preserves biochemical hemostasis ability of the platelets while limiting their flow-driven thrombosis. Meanwhile, the demonstration of force-direction-dependent platelet activation also provides an insight into the biomechanical principles of platelet activation. Coupled with microfluidic technology, it may be possible to control the flow conditions that promote platelet rolling or sliding, enabling further exploration of mechanosensing pathways and analysis of how force direction shapes thrombus architecture.

Current extracorporeal life support treatments rely on anticoagulants, like heparin or citrate, which block coagulation and carry a high bleeding risk. 22% of CRRT patients (53), where the proportion increased to 48% in the sepsis subgroup (54), must undergo anticoagulant-free protocols for safety. Currently, anticoagulant-free treatments require enhanced device anticoagulation and frequent saline flushes. Meanwhile, platelet spherification presented in this work provides an alternative solution to anticoagulation in extracorporeal therapies.

Unlike conventional strategies that suppress the coagulation cascade and inevitably increase bleeding risk, spherification selectively restrains thrombus growth within the dialyzer without blocking the activity of soluble agonists. A low dose of colchicine administered 30 min before treatment was sufficient to induce this morphological switch, within a clinically safe dosing range and without detectable adverse effects. By shifting the approach from systemic anticoagulation to morphology-based modulation, this strategy offers a practical and patient-friendly solution to a long-standing dilemma in anticoagulation-free dialysis. For patients at high risk of bleeding, platelet spherification could enable extracorporeal circulation to be safer and simpler, pointing to a transformative direction for dialysis and other extracorporeal life support. Thus, platelet-morphology-based anticoagulation has practical potential in extracorporeal life support.

## Materials and Methods

All materials and methods are described in detail in *SI Appendix*.

Blood samples are collected from healthy volunteers via venipuncture. These donors refrained from taking platelet-altering medications for at least 2 wk before donation. The samples are collected in vacuum medical anticoagulant tubes containing 3.2% sodium citrate and used within 1 d. Blood samples are collected from healthy volunteers via venipuncture. These donors refrained from taking platelet-altering medications for at least 2 wk before donation. The samples are collected in vacuum medical anticoagulant tubes containing 3.2% sodium citrate and used within 1 d. Informed consent was obtained from all donors and all experiments were performed in compliance with the relevant laws and guidelines approved by the Institutional Review Board (IRB) of Tsinghua University (Approval No. AP18-CHS1). The samples are destroyed after use by relevant laws and regulations.

Methods for platelet isolation, platelet adhesion, platelet fluorescence staining, fabrication and observation of hemostasis microfluidic chip, design of microfluidic chip, platelet shape and related detection, inclined-plane experiment, AFM setting and probes functionalization, AFM data processing, FLIM-based FRET, MD System Setup, SMD by constant force and constant velocity, reversibility assay of platelets, In vitro hemodialysis experiments, ACT testing, and method used to calculate occlusion ratios are detailed in *SI Appendix*.

## Supplementary Material

Appendix 01 (PDF)

Movie S1. Discoid platelets mediate effective hemostasis.

Movie S2. Spherical platelets reduce the thrombus formation.

Movie S3. Discoid platelets slid along the surface.

Movie S4. Spherical platelets roll along the surface.

Movie S5. MD results under tangential traction force.

Movie S6. MD results under normal traction force.

Movie S7. The 50% spherical-platelet condition still achieved hemostasis, but with slower hemostatic kinetics.

Movie S8. Morphological recovery of platelets from a spherical to a discoid shape over time (150× speed).

## Data Availability

MD data have been deposited in GitHub (https://github.com/DrJR2000/MD_Result_a2bb3_PNAS.git) (55). All other data are included in the article and/or supporting information.
