# Supplementary material for: Biomechanical anticoagulation by spherical platelets in extracorporeal systems
Source: Proc Natl Acad Sci U S A. 2026 Mar 30;123(14):e2535113123. doi: 10.1073/pnas.2535113123 (PMC13056125; doi:10.1073/pnas.2535113123)
Supplement: Supplementary file 1 — Appendix 01 (PDF) [file pnas.2535113123.sapp.pdf]

PNAS

Supporting Information for
Biomechanical Anticoagulation by Spherical Platelets in Extracorporeal Systems

Rui Ji, Yongjian Li, Jiang Li, Haosheng Chen

*Haosheng Chen
Email: chenhs@tsinghua.edu.cn

This PDF file includes:

Supporting text
Supplementary Figures S1 to S8
Legends for Movies S1 to S8
SI References

Other supporting materials for this manuscript include the following:

Movies S1 to S8

Supporting Information Text

Platelet Isolation

Platelets are isolated using a MiniSpin centrifuge (MiniSpin, Eppendorf Co.). Blood is centrifuged at 150 g for 10 minutes. The supernatant is centrifuged at 1350 g for 5 minutes to isolate the platelet-rich pellet. The resulting platelet pellet is resuspended in PBS. If needed to inhibit activation, platelets are treated with a solution containing ACD (10%, vol/vol, C3821, Sigma Co.), heparin (10 μM, H8060, Solarbio Co.), PP2 (5 μM, T6266, TargetMol Co.) and SC-99 (5 μM, S23510, Medmol Co.). ACD and heparin are added to inhibit platelet activation by soluble agonists. PP2 and SC-99 are added to maintain $\alpha_{IIb}\beta_3$ in a bent-closed conformation. To induce platelet spherization, 20nM colchicine is added and incubated for 10 minutes to ensure complete reaction. If neutralize the citrate anticoagulation is required, a recalcification solution containing 75 mM $CaCl_2$ and 25 mM $MgCl_2$ in deionized water is prepared. This solution was then mixed with citrated whole blood at a 1:10 (v/v) ratio for recalcification.

Platelet Adhesion

Glass slides are first coated with a 1 μmol/ml dopamine solution (A902400, MACKLIN Co.), prepared in Tris buffer, pH 8.5), and incubated for 24 hours. After three washes with ultrapure water, the platelet suspension is applied to the slides and incubated for 10 minutes. Non-adherent platelets are removed by washing with PBS. If needed to activate platelets, slides are incubated with 10 μM ADP.

Platelet Fluorescence Staining and quantitative analysis of fluorescence intensity

DiOC6 (1%, vol/vol, D273, ThermoFisher Co.) is used to label the endoplasmic reticulum of the platelets. CD62P-PE (1%, vol/vol, 12-0626-82, ThermoFisher Co.) labels activated platelets via P-selectin expression. After washing with PBS, stained platelets are incubated on glass slides for 5 minutes. Fluorescence is observed by the inverted microscope (DMi8, Leica Co.) within 15 minutes to achieve optimal imaging. Each fluorescence intensity value was calculated from an individual platelet. Green fluorescence was first used to delineate the region of each platelet. The yellow fluorescence intensity within each region was then quantified. Finally, all fluorescence data were normalized across samples for comparison.

Fabrication and Observation of Hemostasis Microfluidic Chip

The microfluidic chip for hemostasis modeling is fabricated using soft lithography. A silicon wafer coated with SU-8 photoresist (Microchem Co.) is patterned using a UV lithography system (SF100, Advanced Micro Patterning Co.) to create the mold for microchannels. A 10:1 mixture of PDMS prepolymer and curing agent is poured onto the patterned wafer, degassed for 20 min, and thermally cured at 70°C for 90 min. The cured PDMS microchannels are peeled off, and inlet/outlet holes (1 mm diameter) are punched using a PDMS puncher. The PDMS layer and a glass slide are plasma-treated for 5 min, bonded together, and baked at 105°C for 15 min to complete chip assembly. For collagen coating, 10 μL of type I collagen solution (0.5 mg/mL, Yeasen Biotechnology Co.) is injected into the buffer channel, filling the vertical channel via capillary action at the liquid-air interface. The chip is stored at 4°C for 12 h to allow collagen adsorption onto the vertical channel walls, forming a pro-coagulant layer. Before experiments, the blood flushing channel is rinsed with 2% BSA in PBS for 5 min and incubated at room temperature for 1 h. Fluorescence microscopy (DMi8, Leica Co.) is used to capture bright-field and fluorescence images of the hemostasis microfluidic chip over time, characterizing the temporal growth of different components within the thrombus during the hemostatic process. Each microfluidic experiment is carried out for 20 minutes, or terminated earlier if coagulation is observed. The 50% spherical-platelet condition was generated by mixing untreated blood with colchicine-treated blood at a 1:1 ratio, and the mixture was used within 20 min after preparation.

Design of Microfluidic Chip

The microfluidic chips consist of a buffer flushing channel, a blood flushing channel, and a vertical channel. Both flushing channels are U-shaped with 1 mm diameter inlets/outlets, interconnected by the vertical channel (40 μm wide × 150 μm long) (Fig. S1A and S1B). For collagen coating, 10 μL of collagen solution (0.5 mg/mL) is slowly injected into the buffer flushing channel, where surface tension maintained the liquid-air interface at the connection of the vertical channel and the blood

flushing channel (Fig. S1C). Incubate the chips 12-hour at 4°C to form a pro-adhesive surface on the vertical channel and buffer flushing channel.
During perfusion, both platelet adhesion area within the vertical channel and blood flow rate were monitored. In the case of discoid platelets, a rapid increase in adhesion area was observed within the first 6 minutes, accompanied by a marked decrease in flow rate, indicating effective hemostasis. In contrast, spherical platelets exhibited a slower increase in adhesion and minimal changes in flow rate, suggesting impaired hemostatic function (Fig. S1D and S1E). Additionally, the motion of platelets was analyzed. Only 20% of discoid platelets displayed rolling behavior, whereas 70% of spherical platelets exhibited rolling along the channel surface (Fig. S1F).

Platelet Shape and Related Detection

After inducing platelet spherization with colchicine, a marked increase in spherical platelets formation was observed. Native and colchicine-treated platelets were incubated with an inhibitor cocktail containing ACD (10% v/v, C3821, Sigma), heparin (10 μM, H8060, Solarbio), PP2 (5 μM, T6266, TargetMol), and SC-99 (5 μM, S23510, Medmol), then gently dropped onto dopamine-coated coverslips and allowed to incubate for 30 min. Platelets were fixed with 2.5% glutaraldehyde for 2 h, followed by three PBS washes. Dehydration was performed using graded ethanol solutions (30%, 50%, 70%, 90%, and 100%), each for 15 min. After dehydration, samples were platinum-coated to ensure conductivity and imaged using a field emission scanning electron microscope (Quanta 200 FEG, FEI Co.). Native platelets exhibited a flattened morphology, whereas colchicine-treated platelets appeared spherical (Fig. S2A and S2B).

Rod-sliding Experiments

Acrylic rectangular (10×5 mm) and round (diameter 8 mm) rods are used in the experiments. Glass sheets (0.1mm thickness) are processed as described in the "platelet adhesion" section, where adherent platelets are stained with DiOC6 and CD62P-PE. The robs are washed three times with ultrapure water, incubated with 0.1 mg/mL RGDs for 30 minutes, and then washed three times with ultrapure water and stored in PBS. The experiment is conducted within 6 minutes of processing and slide 20 times per minute. The results are observed using an inverted microscope.

Inclined-plane Experiments

Acrylic rectangular (10×5 mm) and round (diameter 8 mm) rods are used in inclined-plane experiments. Glass sheets (0.1mm thickness) are incubated with 0.1 mg/mL RGDs for 30 minutes, then washed three times with ultrapure water, and finally stored in PBS.
The robs are first coated with a 1 mol/ml dopamine solution (A902400, MACKLIN Co.), prepared in Tris buffer, pH 8.5), and incubated for 24 hours. After three washes with ultrapure water, the platelet suspension is applied to the rods and incubated for 10 minutes. Non-adherent platelets are removed by washing with PBS. Adherent platelets are stained with DiOC6 and CD62P-PE. By increasing the tilt angle of the slide, the round rods transitioned from pure rolling to a combined rolling-sliding motion. As the tilt angle increased, the sliding fraction rose accordingly. Three tilt conditions were selected, corresponding to sliding fractions of 0%, 20%, and 50%. The experiment is conducted within 6 minutes of processing and slide 20 times per minute. The results are observed using an inverted microscope (Fig. S3).

AFM Setting and Probes

AFM measurements are conducted using the biological scanning probe microscope (CSPM5500B, Being Co.). Probes with a spring constant of 0.01 N/m and a resonant frequency of 11.0 kHz (qp-scont, NANOSENSORS Co.) are used for tangential scanning. The probes have geometric dimensions of 125 μm (length), 34 μm (width), and 0.35 μm (thickness) with a tangential force coefficient of 10. For normal scanning, probes (SD-R30-CONTAuD, NANOSENSORS Co.) with a spherical tip (30nm radius) have a spring constant of 0.11 N/m and a resonant frequency of 25.0 kHz.

RGDs Probe Functionalization

Probes are first treated by the oxygen plasma treatment for 30 minutes to increase hydrophilicity, then incubated with 1 mg/mL NHS-PEG-COOH with a molecular weight of 3400 Da (X-GF-0161-3.4k, Xiankaixin Co.) at room temperature for 2 hours. After washing three times with ultrapure

water, probes are activated with 0.4 M 1-ethyl-3-(3-dimethylaminopropyl) carbodiimide (EDC) (22981, ThermoFisher Co.) and 0.1 M N-hydroxysuccinimide (NHS) (24500, ThermoFisher Co.) at room temperature for 1 hour. Then, wash the probes three times with ultrapure water. The probes are incubated with 0.1 mg/mL RGDs (91037-65-9, ChinaPeptides Co.) for 30 minutes, followed by washing and incubation with ethanolamine (E103806, Aladdin Co.) for 30 minutes to prevent nonspecific binding. Functionalized probes are used in one day or stored at 4°C for up to two days.

Protein Probe Functionalization

The probes are plasma-treated for 15 minutes and then incubated in a 1% (v/v) alcohol solution of 3-aminopropyltriethoxysilane (APTES) for 30 minutes. After flushing with ultrapure water, probes are reacted with 10% glutaraldehyde in the aqueous solution for 1 hour. Wash the probes with ultrapure water, then respond with protein (bovine serum albumin, collagen, or fibrinogen) solution (25 μg/mL) for 1 hour. After thorough washing, probes are stored in a buffer solution at 4 °C.

Dissociation Experiments

For vertical force spectroscopy, AFM is used to measure the interaction forces between integrin $\alpha_{IIb}\beta_3$ and ligands using functionalized probes. Each force curve consisted of 200 data points, and each point was avenged five times. Lateral force microscopy measurements included 1024 data points per curve, with each point avenged twice. The scan rate is 0.8 Hz, and the scale is 200×200 nm^2.

AFM Data Processing

For tangential force curves, the point preceding the force jump is considered the origin, and the 150 data points before and the 50 data points after this point are recorded. The last sampling point is the origin of vertical force curves, and all 200 data points are recorded. The data is filtered with a moving average of 7 to reduce background noise.

Contact and Tapping Scanning of Platelets

Platelet scanning is performed using the AFM (CSPM5500B, Being Co.) in combination with the inverted microscope (DMi8, Leica Co.) to determine the relative position of the probe and the adherent platelets. The scans are performed with a precision of 64x64, at a scanning rate of 2 Hz and a scan width of 10,000 nm. Fluorescence changes are observed after every six scans (approximately 3 minutes).

Fluorescence Lifetime Imaging Microscopy (FLIM)–based FRET

FLIM data were acquired using a Leica STELLARIS 8 FALCON confocal microscope integrated with LAS X software, optimized for lifetime measurement. FLIM-FRET was performed by exciting the donor fluorophore at its optimal wavelength and collecting emission with appropriate spectral filters. Lifetime decays were transformed into phasor space, and the universal semicircle was used to discriminate single-exponential and multicomponent lifetimes on a pixel-by-pixel basis. FRET efficiencies were estimated by comparing donor lifetime distributions in the presence and absence of acceptor, independent of intensity and fluorophore concentration. All lifetime analyses, phasor plot generation, and cluster gating were performed in LAS X's phasor analysis module, ensuring robust separation of FRET-active regions and quantitative calculation of apparent FRET efficiency.

Supplementary Data of AFM Experiments

Although AFM does not allow flexible modulation of rolling-to-sliding ratios like plane-slider experiments, it serves as a complementary tool to validate the contribution of tangential force to integrin activation. AFM continuously stimulated the adhered platelets in contact or tapping modes (Fig. S6A). The fluorescence intensity peaking at 9 min, indicating that tangential forces activated the platelets. Tapping mode showed no significant activation markers, indicating low platelet sensitivity to normal forces.

Two peaks were observed before the force jump during the tangential force scan on inactive $\alpha_{IIb}\beta_3$. The amplitudes of the two peaks are 2.2 and 2.15 times the standard deviation of the baseline noise, respectively (Fig. S6B). According to z-test criterion, a signal is considered statistically significant if its amplitude exceeds 1.96 times the standard deviation of the noise. Therefore, both peaks can be regarded as statistically significant events preceding $\alpha_{IIb}\beta_3$ unfolding.

After incubating platelets with ADP for 20 minutes to activate them, the platelets' tangential force curves are measured. The rapture force of the activated integrin reaches 91.3 ± 9.5 pN and the peaks previously seen in the inactive integrin did not appear (Fig. S6C).

In acceptor photobleaching FRET analysis, the DiI fluorescence intensity before bleaching is denoted as I_{pre}, and the intensity after bleaching is denoted as I_{aft}. The FRET efficiency η_{FRET} is

calculated using the following equation: $\eta_{FRET} = (I_{aft} - I_{pre}) / I_{aft}$. RGDs are used to block the integrin binding site. After RGDs incubation, the FRET effect remained low regardless of whether the platelets are exposed to tangential or normal forces (Fig. S6D).

Molecular Dynamics (MD) System Setup

The MD simulations are performed employing the GROMACS molecular dynamics package. The CHARMM36 force field (1) and the TIP3 water model (2) describe all systems. The structure of integrin αIIbβ3 is available at the RCSB Protein Data Bank (PDB: 9DEQ) (3). We removed all small molecules except the ligand and ions near the binding site, and generated the membrane-embedded system and initial model using CHARMM-GUI Membrane Builder (4). To approximate platelet plasma-membrane lipid asymmetry, we constructed an asymmetric bilayer with POPC in the outer leaflet and POPS in the inner leaflet, consistent with the enrichment of aminophospholipids such as PS on the cytosolic leaflet in resting platelets (5). Phosphorus atoms were restrained individually to their original position by springs with a spring constant of 1000 kJ/mol/nm2 to avoid translational movement induced by the pulling (6). The box is extended by 100 Å along the traction direction to ensure it does not exceed the space during simulations. Extend the rest direction by 15Å to ensure that periodic boundaries do not affect αIIbβ3. The structure is solvated with a NaCl concentration of 0.15 M.

Energy Minimization and Equilibration

The steepest descent algorithm relaxes through the structure. Then, a 100-ps NVT equilibration is conducted to maintain the temperature at 300 K using Langevin dynamics. The pressure of the system is maintained at 1 atmosphere using the Langevin piston during a 100-ps NPT equilibration. The time step is set to 2 fs. A cut-off distance of 1.2 nm is used for nonbonded interactions. Particle mesh Ewald (PME) is employed for electrostatic calculations. The SHAKE algorithm is applied to constrain the hydrogen bond lengths. The SHAKE algorithm is also used to constrain the bond lengths between heavy atoms and hydrogen atoms. During equilibration, update the van der Waals interactions at every step and electrostatic interaction at every two steps.

SMD by Constant Force

In the steered molecular dynamics (SMD), temperature and pressure controls are turned off, which avoids unnecessary external forces caused by balance. The tail residues GLN950-CYS959 (GLN: Glutamine, CYS: Cysteine and 950 denotes residue number) of the α subunit and the tail residues THR603-LYS612 (THR: Threonine, LYS: Lysine) of the β subunit are fixed to keep the integrin tail stable. During the constant force traction, ligand tirofiban is chosen as the driving molecule. The pulling force is measured as 1 kcal·Å/mol, which is approximately 70pN (100 pN equivalent to 1.44 kcal·Å/mol). Perform two simulations, one with traction force tangential to the integrin $\alpha_{IIb}\beta_3$ and the other normal to the integrin. Each simulation lasted for 10 ns.

To further characterize the conformational change of integrin $\alpha_{IIb}\beta_3$ under force, the displacement of the traction point D_F along the force direction and the interaction energies between key domains were analysed. Under normal force, D_F increased to 9 nm and then plateaued, suggesting a rapid but limited rotation of the traction point during the conformational change. In contrast, tangential force resulted in a steady increase in D_F up to 5 nm (Fig. S7C and S7D). Interaction energies Es between the Hybrid domain and I-EGF3/I-EGF4 were examined to evaluate domain separation. Under normal force, Es decreased but did not reach 0 kJ/mol, indicating incomplete dissociation. In contrast, tangential force fully disrupted the interactions, with Es dropping to 0 kJ/mol (Fig. S7E). This suggests that the rotation induced by normal force is insufficient for $\alpha_{IIb}\beta_3$ unfolding. Additionally, the interaction energy Er between calf-1 and calf-2 was analysed. Under normal force, E_r increased from -130 kJ/mol to -60 kJ/mol, indicating compression between these domains (Fig. S7F). It is the reason why the observed plateau in D_F and the limited unfolding under normal force.

SMD by Constant Velocity

The constant-force SMD allowed us to test whether the integrin can be mechanically activated under sustained force input. In contrast, constant-velocity SMD provided instantaneous traction forces required for the conformational changes, enabling comparison with AFM experiments.

In the constant velocity pulling simulation, ligand is also the driving molecule. The spring constant between the driving SMD and dummy atoms is set to 7 kcal·Å/mol. The average tangential pulling velocity during the 'Separation I3' stage was 0.095 nm/ns, while the average velocity during the 'Rotation' stage in normal pulling was 1.23 nm/ns. To eliminate the influence of pulling speed on

instantaneous force, the normal pulling velocity was adjusted to match the tangential one, 0.095 nm/ns. For each simulation, the total pulling distance was set to 10 nm. which is close to the constant force pulling results. Umbrella sampling is used for constant-speed traction, and the simple harmonic force constant is 1000 kJ/mol*nm^2.

Reversibility Assay of Platelets

After the *in-vitro* experiments, platelets were re-pelleted as in "Platelet isolation", supernatant was discarded, and PBS was added to remove colchicine. Samples were taken every 10 min and imaged on an inverted microscope (DMi8, Leica).

***In-vitro* Hemodialysis Experiments**

Porcine blood preserved with 3.2% sodium citrate was used within 24 h at room temperature. Colchicine (20 nM) was added and incubated at 37 °C for 30 min. The dialysis circuit was assembled using components from a commercial single-use blood tubing set (BLS-101-HA, Hanahao Co.), including selected connectors, portions of the tubing, and the venous canister. Prior to circulation, the circuit was preflushed with normal saline and thoroughly de-aired to remove residual air bubbles. Calcium solution was then added to neutralize citrate, and the blood was quickly introduced into the loop to start circulation. Inlet pressure and flow were recorded every 2 min. ACT was measured every 5 min from a sampling port (200 µL each). After the circulation stopped, the circulation was flushed with saline for 5 min.

ACT Testing

Clotting time was measured using a kaolin-phospholipid suspension-based commercially available reagent kit (Leagene Co.). Because our samples were citrate-anticoagulated, we performed a standardized recalcification step to fully restore Ca^{2+} to physiological levels prior to timing. All groups, including the sterile porcine whole-blood control, were processed with the identical recalcification and assay workflow.

Method Used to Calculate Occlusion ratios

To quantify dialyzer occlusion, we perfused the dialyzer with fluorescein sodium solution after the experiment. Regions of hollow fibers that were occluded did not perfuse and therefore were not fluorescently stained. The dialyzer was imaged under fluorescence, and fluorescence area/intensity was quantified using ImageJ. We define the patency ratio as: $P_{occlusion}=A_{post}/A_{new}$, where A_{post} is the fluorescent area measured in the dialyzer after the experiment and A_{new} is the fluorescent area of an unused dialyzer imaged under the same settings.

Figures

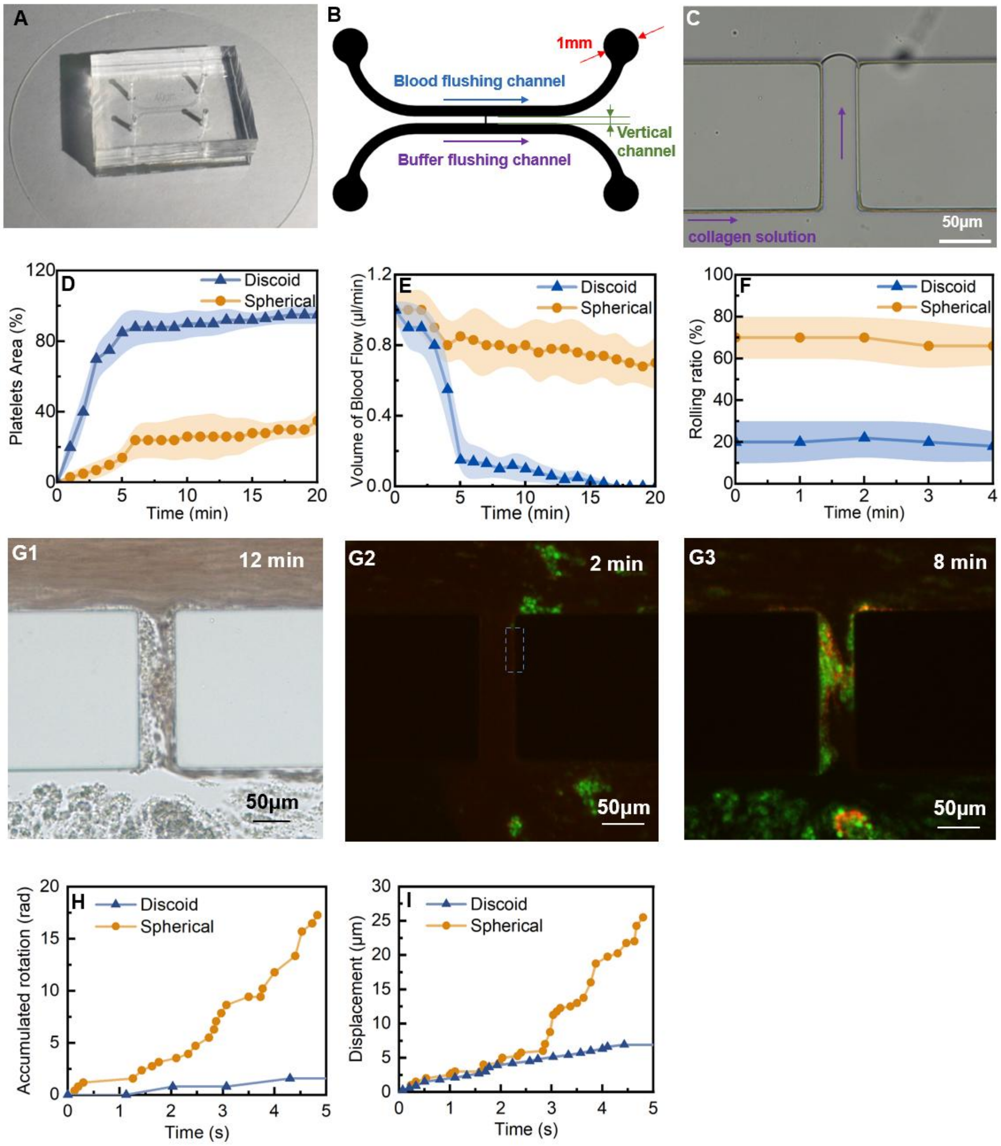

Fig. S1. Hemostasis microfluidic chips design. (A) Physical image of the chip. (B) Schematic of microchannels with 1 mm diameter inlets/outlets, arrows indicate flow direction. (C) Collagen solution incubation in buffer channel, maintained at vertical channel. (D) Platelet adhesion area changes at the vertical channel during the perfusions. (E) Volume of blood flow changes through the vertical channel during the perfusions. (F) Proportion of rolling motion through the vertical channel during the perfusions. (G) Hemostasis testing of the 50% spherical platelet group was performed in microfluidic devices. (H,I) The accumulated rotational angle and displacement of the discoid or spherical platelet, which were quantified based on Movies S3 and S4.

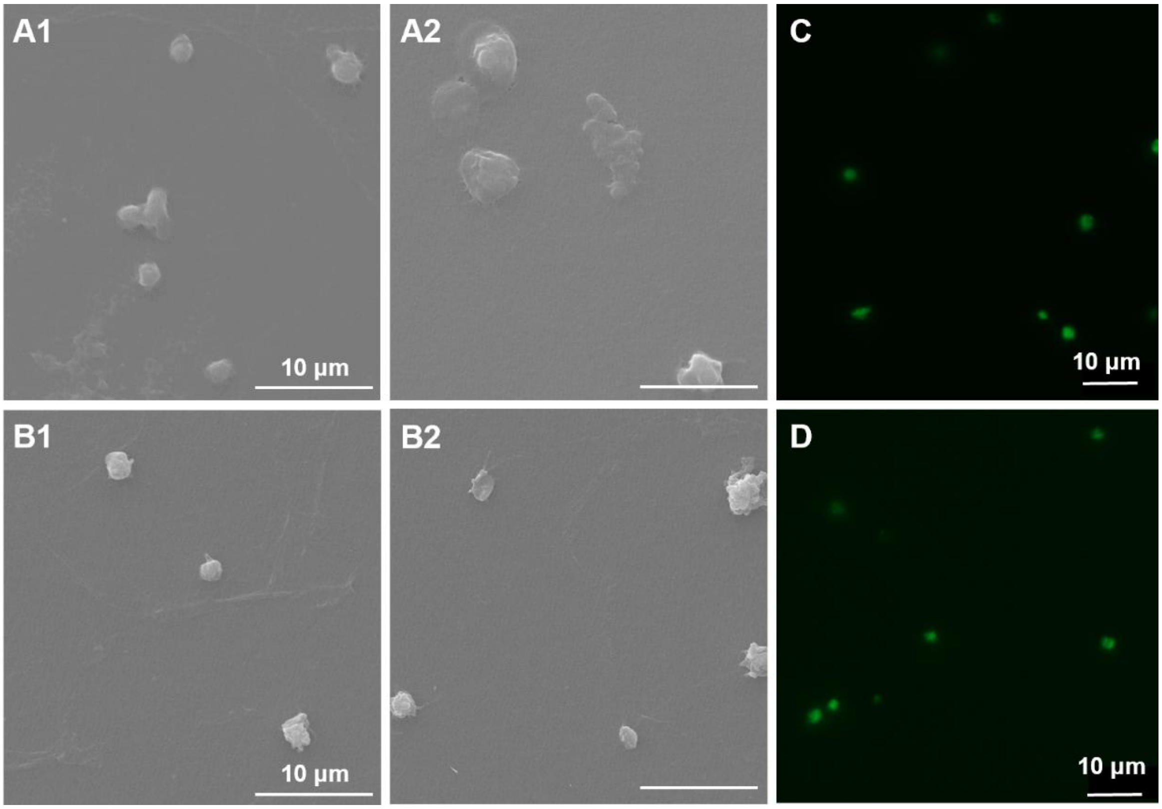

Fig. S2. Platelet spherocytosis treatments and results of platelet adhesion assays. (A) Electron microscopy images of native platelets. (B) Electron microscopy images of colchicine treated platelets. (C) Fluorescence results of native platelets adhesion. (D) Fluorescence results of treated platelet adhesion.

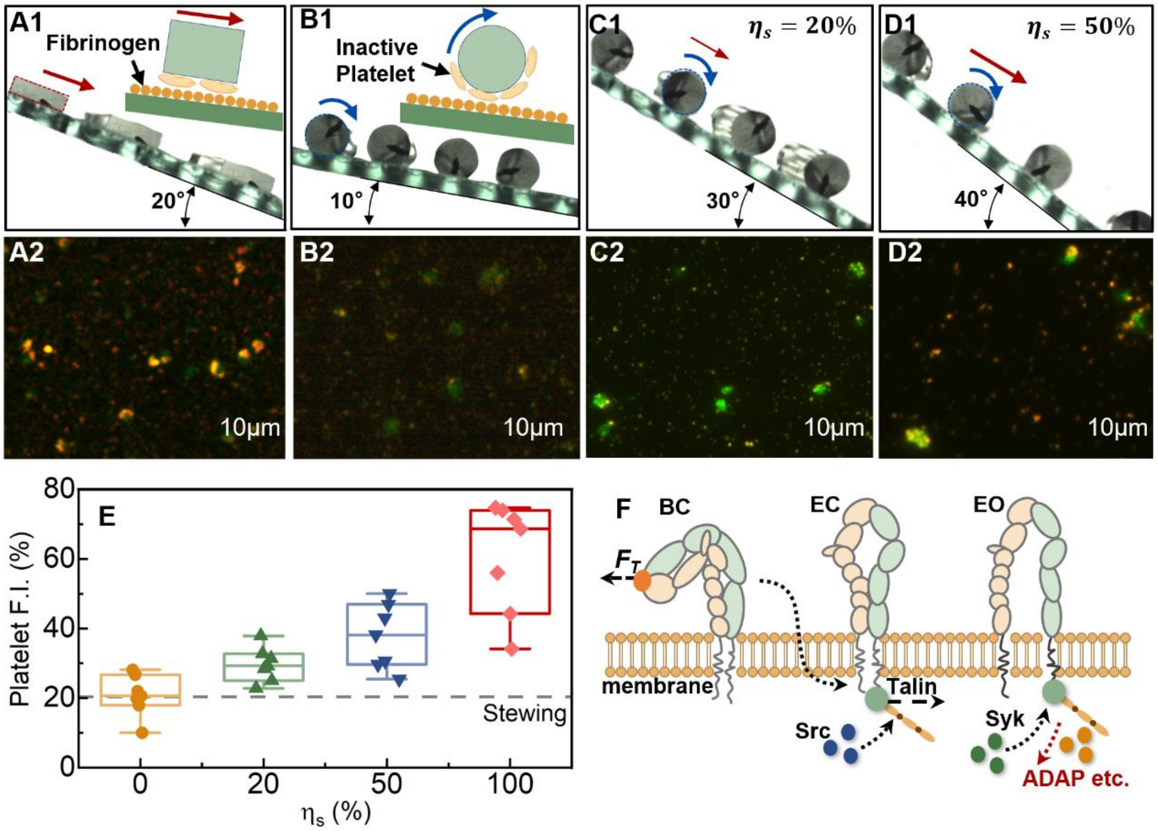

Fig. S3. Experiments on force stimulation triggered platelet activation. (A1) The inclined-plane experiment to test the platelet activation under sliding conditions, and (A2) illustrates the yellow fluorescence from the activated platelets. (B1-D1) The inclined-plane experiment to test the platelet activation under sliding-rolling condition. (B2-D2) The corresponding fluorescence of platelets in sliding-rolling motions. (E) The fluorescence intensity (F.I.) of platelets changed with the sliding-rolling ratios. (F) The schematics of the platelet activation pathway induced by the unfolding of $\alpha_{IIb}\beta_3$.

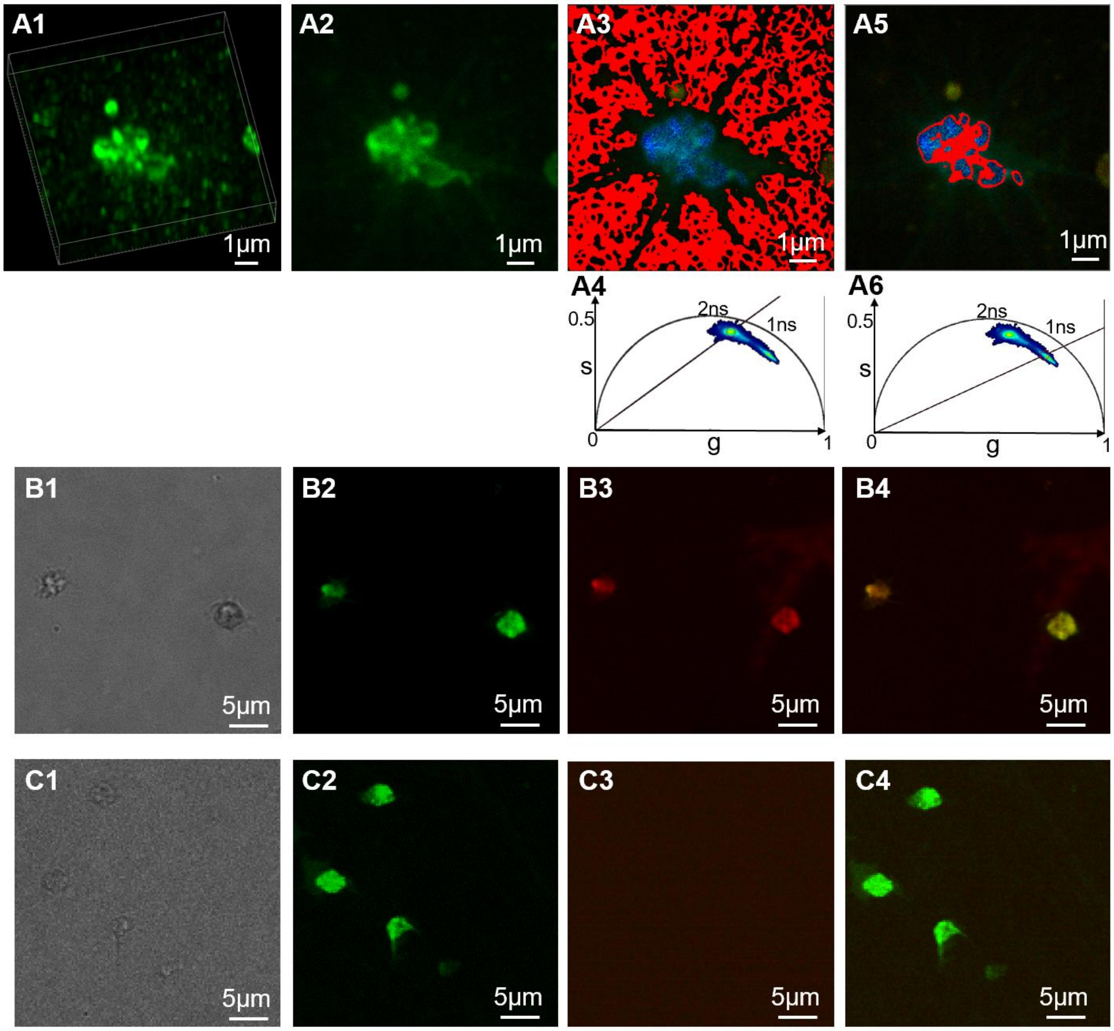

Fig. S4. Supplementary data for fluorescent labeling. (A1) Three-dimensional z-stack imaging of platelets. (A3-6) Membrane staining with DiO. Below the fluorescence image (A4 and A6) is the corresponding fluorescence lifetime phasor diagram. In the fluorescence lifetime phasor diagram, the position and spread of the phasor cluster reflect the relative contributions of different lifetime populations. Phasor analysis resolved two components: one corresponding to background fluorescence from DiO that was not membrane-incorporated, and a second component corresponding to membrane-inserted DiO. The latter mapped predominantly to the cell boundary, forming an irregular band-like pattern along the platelet membrane. (B) Bright-field, fluorescence imaging, and colocalization analysis of platelets simultaneously labeled with DiO and Rhodamine B-RGDS. (C) After treatment of platelets with tirofiban, bright-field, fluorescence imaging, and colocalization analysis were performed for platelets labeled with DiO and Rhodamine B-RGDS. Rhodamine B-RGDS failed to label platelets under this condition.

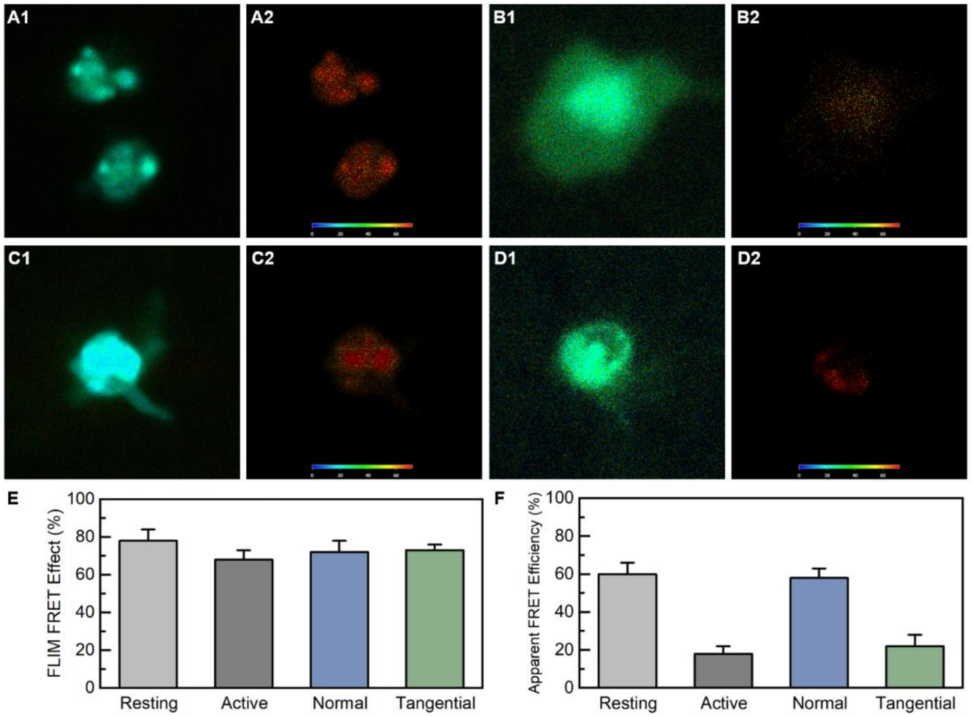

Fig. S5. Supplementary data for FLIM-FRET. (A) Fluorescence image of resting platelets and the FRET efficiency map. (B) Fluorescence image of activated platelets and FRET efficiency map. (C) Fluorescence image of platelets after normal mechanical stimulation and FRET efficiency map. (D) Fluorescence image of platelets after tangential mechanical stimulation and FRET efficiency map. (E) FLIM-FRET efficiency under different conditions, representing the mean FRET efficiency within the regions where FRET is detected. (F) Apparent FRET efficiency under different conditions, representing the surface-averaged FRET efficiency across the platelet.

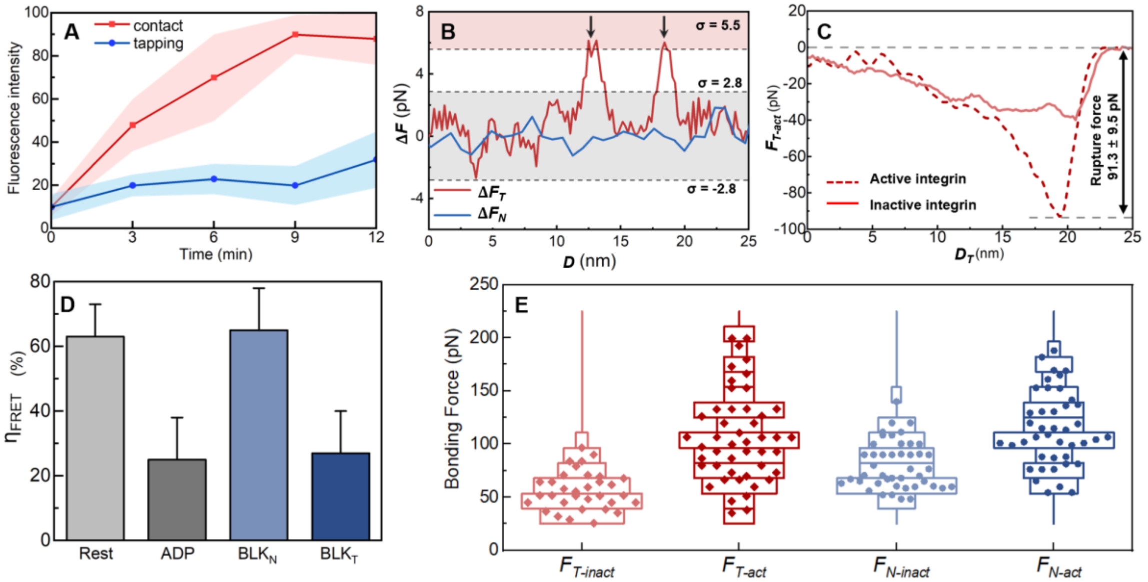

Fig. S6. Supplementary data for AFM experiments. (A) The fluorescence intensity-scanning time curve of contact and tapping mode. (B) The distribution map of detected peaks, with the horizontal axis representing displacement *D* under sliding and rolling conditions. It shows that the difference from the baseline ΔF of two peaks is quantitatively higher than the standard deviation of the noise σ_n. (C) The bonding forces of activated and inactivated $\alpha_{IIb}\beta_3$ under sliding conditions. For activated $\alpha_{IIb}\beta_3$, there is no peak on the curve, and the jump amplitude 91.3 ± 9.5 pN is larger than inactivated $\alpha_{IIb}\beta_3$. (D) FRET efficiency η_{FRET} under various conditions: Stew. (Stewing as negative control), ADP (treated with ADP as positive control), BLK_N (normal force without inhibitor) and BLK_T (tangential force without inhibitor). (E) Distribution of activated and inactive integrins' binding forces under tangential and normal scans.

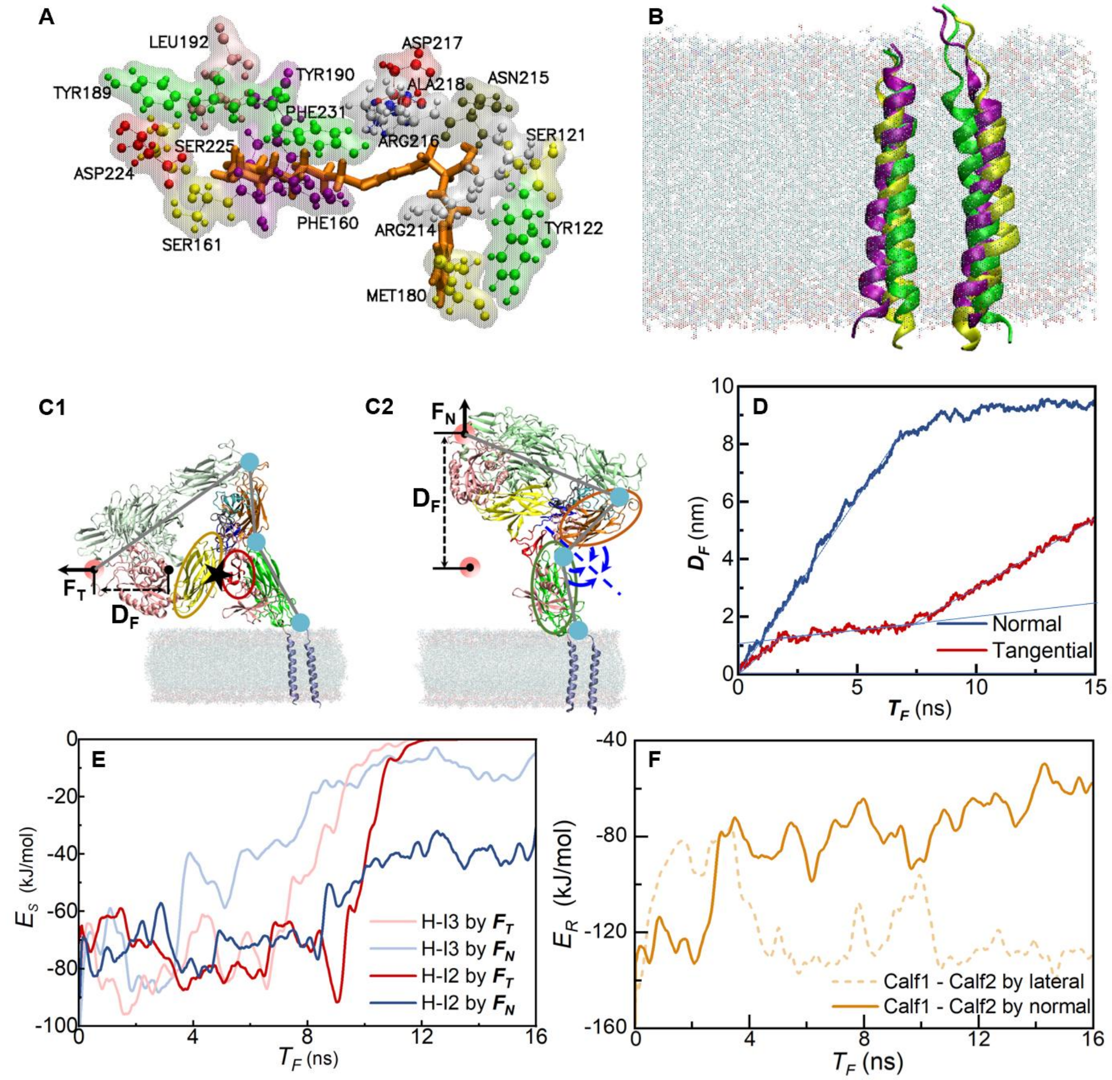

Fig. S7. Supplementary data for MD simulation. (A) The inserted ligand and surrounding integrin residues. (B) Conformations of the integrin cytoplasmic tails. Yellow indicates the initial conformation; green indicates the conformation after tangential pulling; and purple indicates the conformation after normal pulling. (C) Under tangential force, D_F represents the lateral displacement of the traction point, whereas under normal force, D_F represents its vertical displacement. (D) Traction time T_F -traction distance D_F curve under tangential and normal forces. (E) Interaction energy E_S -traction time T_F curve between Hybrid and I-EGF3 (H-I3) or between Hybrid and I-EGF4 (H-I4). (F) The interaction energy E_R -traction time T_F curve between Calf-1 and Calf-2 on the $\alpha_{IIb}\beta_3$. The solid line represents the result pulled by tangential force and the dashed line is normal.

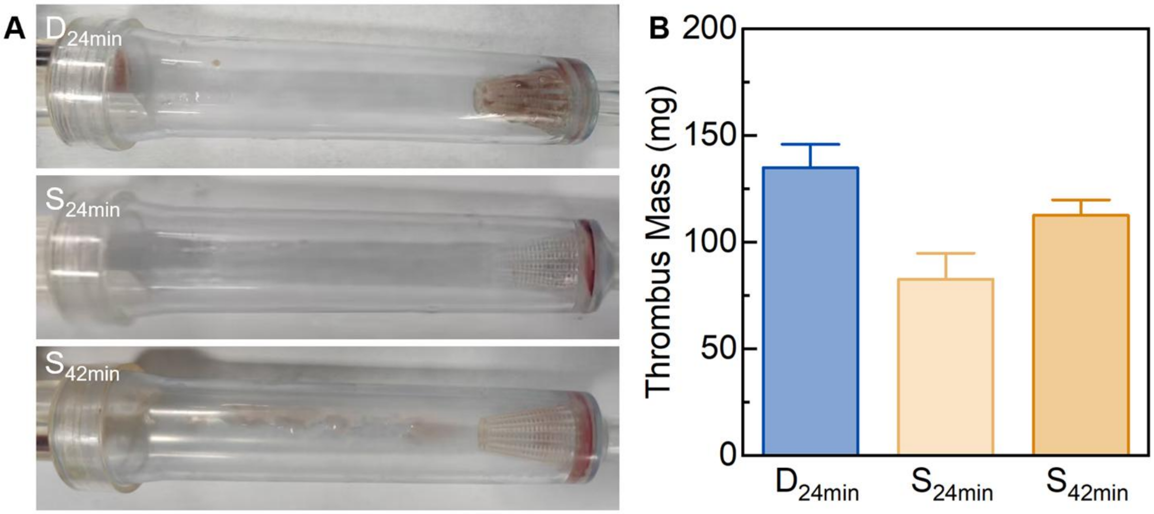

Fig. S8. Collect free blood clots using a v venous canister. (A) Images of thrombosis collected by venous canister after dialysis experiments (D represents discoid platelets, S represents spherical platelets, 24min indicates the circulation time). (B)The quality of blood clots collected from venous canister after each dialysis test.

Movie S1 (separate file). Discoid platelets mediate effective hemostasis.

Movie S2 (separate file). Spherical platelets reduce the thrombus formation.

Movie S3 (separate file). Discoid platelets slid along the surface.

Movie S4 (separate file). Spherical platelets roll along the surface.

Movie S5 (separate file). MD results under tangential traction force.

Movie S6 (separate file). MD results under normal traction force.

Movie S7 (separate file). The 50% spherical-platelet condition still achieved hemostasis, but with slower hemostatic kinetics.

Movie S8 (separate file). Morphological recovery of platelets from a spherical to a discoid shape over time (150× speed).

SI References

1. R. B. Best et al., Optimization of the additive CHARMM all-atom protein force field targeting improved sampling of the backbone ϕ, ψ and side-chain χ1 and χ2 dihedral angles. Journal of chemical theory and computation **8**, 3257-3273 (2012).
2. W. L. Jorgensen, J. Chandrasekhar, J. D. Madura, R. W. Impey, M. L. Klein, Comparison of simple potential functions for simulating liquid water. The Journal of chemical physics **79**, 926-935 (1983).
3. dair BD, Field CO, Alonso JL, et al. Platelet integrin αIIbβ3 plays a key role in a venous thrombogenesis mouse model. Nat Commun **15**:8612 (2024).
4. Jo S, Lim JB, Klauda JB, Im W, CHARMM-GUI Membrane Builder for mixed bilayers and its application to yeast membranes. Biophys J **97**:50-58 (2009).
5. Lhermusier T, Chap H, Payrastre B, Platelet membrane phospholipid asymmetry: from the characterization of aminophospholipid translocases to the identification of floppases. J Thromb Haemost, **9**(10):1883–1891. (2011).
6. K. Wang, Z. Li, Steered molecular dynamics simulation of force triggering the integrin αIIbβ3 extension via its ligand. The European Physical Journal Special Topics **232**, 2773-2781 (2023).
